# Formulation and Scale-up of Delamanid Nanoparticles via Emulsification for Oral Tuberculosis Treatment

**DOI:** 10.1021/acs.molpharmaceut.3c00240

**Published:** 2023-08-14

**Authors:** Nicholas J. Caggiano, Madeleine S. Armstrong, Joanna S. Georgiou, Aditya Rawal, Brian K. Wilson, Claire E. White, Rodney D. Priestley, Robert K. Prud’homme

**Affiliations:** † Department of Chemical and Biological Engineering, Princeton University, Princeton, New Jersey 08544, United States; ‡ Mark Wainwright Analytical Centre, University of New South Wales, Sydney, NSW 2032, Australia; ⊥ Department of Civil and Environmental Engineering, Princeton University, Princeton, New Jersey 08544, United States; ∥ Andlinger Center for Energy and the Environment, Princeton University, Princeton, New Jersey 08544, United States; # Princeton Materials Institute, Princeton University, Princeton, New Jersey 08544, United States

**Keywords:** encapsulation, nanoparticle, emulsion, tuberculosis, oral delivery

## Abstract

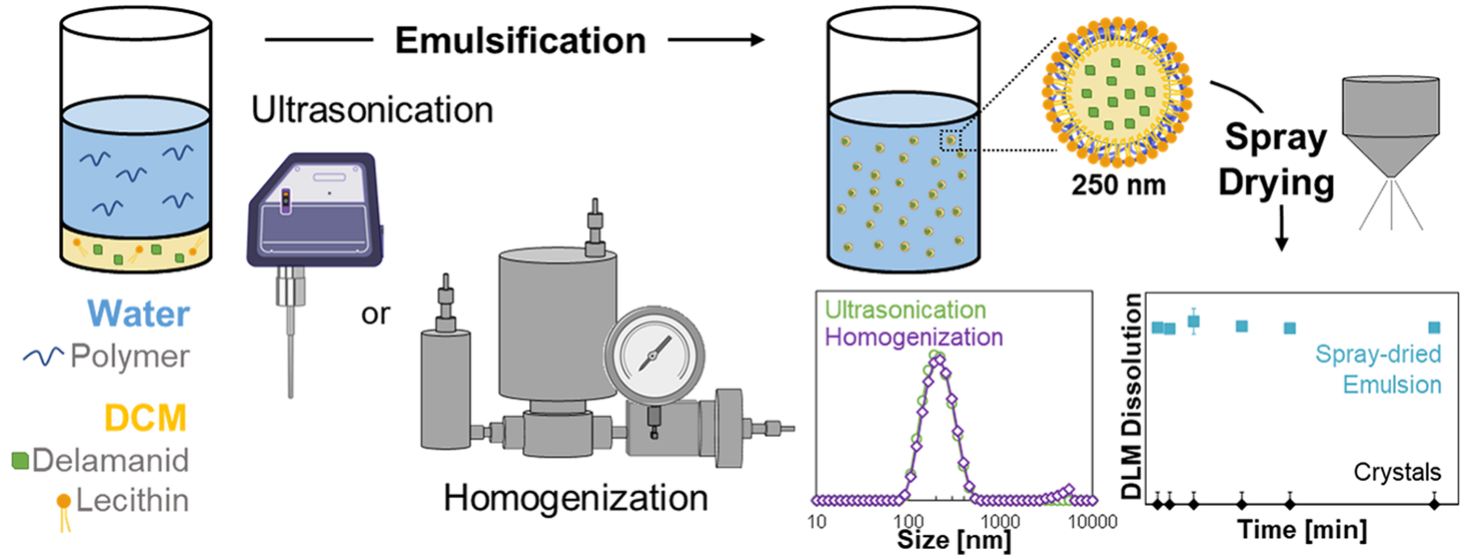

Delamanid (DLM) is a hydrophobic small molecule therapeutic used to treat drug-resistant tuberculosis (DR-TB). Due to its hydrophobicity and resulting poor aqueous solubility, formulation strategies such as amorphous solid dispersions (ASDs) have been investigated to enhance its aqueous dissolution kinetics and thereby improve oral bioavailability. However, ASD formulations are susceptible to temperature- and humidity-induced phase separation and recrystallization under harsh storage conditions typically encountered in areas with high tuberculosis incidence. Nanoencapsulation represents an alternative formulation strategy to increase aqueous dissolution kinetics while remaining stable at elevated temperature and humidity. The stabilizer layer coating the nanoparticle drug core limits the formation of large drug domains by diffusion during storage, representing an advantage over ASDs. Initial attempts to form DLM-loaded nanoparticles via precipitation-driven self-assembly were unsuccessful, as the trifluoromethyl and nitro functional groups present on DLM were thought to interfere with surface stabilizer attachment. Therefore, in this work, we investigated the nanoencapsulation of DLM via emulsification, avoiding the formation of a solid drug core and instead keeping DLM dissolved in a dichloromethane dispersed phase during nanoparticle formation. Initial emulsion formulation screening by probe-tip ultrasonication revealed that a 1:1 mass ratio of lecithin and HPMC stabilizers formed 250 nm size-stable emulsion droplets with 40% DLM loading. Scale-up studies were performed to produce nearly identical droplet size distribution at larger scale using high-pressure homogenization, a continuous and industrially scalable technique. The resulting emulsions were spray-dried to form a dried powder, and *in vitro* dissolution studies showed dramatically enhanced dissolution kinetics compared to both as-received crystalline DLM and micronized crystalline DLM, owing to the increased specific surface area and partially amorphous character of the DLM-loaded nanoparticles. Solid-state NMR and dissolution studies showed good physical stability of the emulsion powders during accelerated stability testing (50 °C/75% RH, open vial).

## 1 Introduction

Tuberculosis (TB) is a bacterial infection of the lung caused by *Mycobacterium tuberculosis*,^1^ which leads to more deaths annually than any other infectious disease (surpassed only by SARS-CoV-2 in 2020). In 2021, there were an estimated 10.6 million global cases of active TB disease, of which 1.6 million were fatal.^2^ Importantly, many cases of tuberculosis are latent and asymptomatic, remaining dormant until active TB disease develops; an estimated 1.7 billion people (23% of the world’s population) were infected with tuberculosis in 2018, including both active and latent cases.^3^

Active TB disease is treated primarily with combination therapy, commonly including a 4 to 6 month course of first-line oral antibiotics isoniazid and rifampin.^4^ However, the rise of drug-resistant tuberculosis (DR-TB), which is resistant to at least rifampin, has necessitated the investigation of alternative treatment regimens. In addition to existing second- and third-line treatments, new nitroimidazole compounds have recently been developed to treat the estimated 500 000 annual cases of DR-TB.^5^ Delamanid and pretomanid are two such compounds and were approved in the European Union in 2014 and 2020, respectively.^6−9^

Delamanid (DLM) is a hydrophobic small molecule (MW = 534 Da, log *P* = 6.1) with limited aqueous solubility (<0.017 mg L^–1^) and a high melting point (*T*_m_ = 196 °C).^6,10^ To enhance the solubility of DLM and improve its oral bioavailability, the current commercialized formulation (trade name Deltyba) is an amorphous solid dispersion (ASD) of delamanid in hydroxypropyl methylcellulose phthalate (HPMCP).^6^ However, storage stability can be a concern for ASD formulations, especially in regions with an elevated temperature and humidity. Plasticization of the ASD polymer matrix caused by heat and moisture can result in drug-polymer phase separation and drug crystallization, which can lead to reduced solubility and bioavailability.^11,12^ As moisture barrier packaging and environmentally controlled storage are cost prohibitive for global heath applications, it would be advantageous to develop a DLM formulation that enhances drug dissolution but is also stable under harsh storage conditions. Recent work has investigated the use of various salt forms of DLM in ASDs to limit drug recrystallization.^13^

Nanoencapsulation represents another formulation strategy for enhancing drug dissolution and bioavailability, while addressing the stability limitations of ASDs. The high specific surface area of the nanoencapsulated drug drives the rapid drug dissolution. Depending on the time scale of nanoparticle self-assembly, the drug may be isolated in its amorphous state, which has higher thermodynamic solubility as compared to the crystalline state. When exposed to elevated temperature and humidity, the stabilizer layer around the nanoparticle drug core limits the formation of large drug domains by diffusion. Thus, even in the case of drug crystallization, the specific surface area of the nanometer-sized drug domains is maintained and the dissolution enhancement performance is retained.

Flash NanoPrecipitation (FNP) is a scalable technique for producing core–shell nanoparticles via rapid antisolvent-induced precipitation and self-assembly.^14^ It has been applied to a wide range of therapeutics, including hydrophobic small molecules and later hydrophilic species via hydrophobic ion pairing. Despite the investigation of process parameters (e.g., solvent, supersaturation ratio, mixing geometry) and formulation parameters (e.g., stabilizer, ion pairing), attempts to nanoencapsulate DLM via FNP were unsuccessful. While NPs were initially formed, they aggregated rapidly. Sufficient time stability (at least 4 h) is needed to enable subsequent processing steps such as concentration (via tangential flow filtration) and spray drying. It is thought that the trifluoromethyl groups of the DLM molecule, due to the fluorous effect,^15−17^ or nitro groups, due to zwitterionic hydration, prevented attachment of the hydrophobic groups of the stabilizing polymer onto the solid drug core formed during the precipitation process. To circumvent the observed drug-stabilizer incompatibility, we investigated emulsification as a method to prepare DLM-loaded nanoparticles while avoiding the formation of a solid drug core. Keeping DLM dissolved in a water-immiscible dispersed phase during emulsification enabled formation of stable nanosized emulsion droplets. The hydrophobic groups of the stabilizer remained anchored on the surface of the nonpolar organic solvent emulsion drop. During subsequent rapid spray drying, the stabilizing shell was immobilized and dried to form a solid drug product. Emulsification has been widely investigated as a pharmaceutical formulation technique, and its scalability makes it attractive for use at industrial scale.^18−22^

Low-cost, naturally derived amphiphilic stabilizers were investigated as emulsifiers, including functionalized cellulosic polymers and l-α-lecithin. Hydroxypropyl methylcellulose (HPMC), hydroxypropyl methylcellulose phthalate (HPMCP), and hydroxypropyl methylcellulose acetate succinate (HPMCAS) are functionalized semisynthetic cellulosic polymers. HPMC and HPMCAS have molecular weights on the order of 20 000 Da,^23,24^ while HPMCP HP-50 is on the order of 40 000 Da.^25^l-α-Lecithin is a soybean-derived mixture of amphiphilic phospholipids comprised of >94% phosphatidylcholine and <2% triglycerides, with a nominal molecular weight of 750 Da. These stabilizers were chosen as a starting point based on extensive prior experience using lecithin and HPMCAS as stabilizers for nanoparticles prepared by FNP.^26−28^

Formulation development revealed that these naturally derived stabilizers could produce nanosized DLM-loaded emulsion droplets. However, the larger, slower diffusing cellulosic polymers resulted in larger droplet sizes than small molecule lecithin. While lecithin can be an effective emulsifier on its own, the elevated temperature used during spray drying plasticizes the lecithin layer, causing aggregation and the fusion of emulsion droplets during drying. Thus, we found that a combination of lecithin and cellulosic stabilizers was effective at producing size-stable ∼250 nm emulsion droplets that were robust enough to be spray dried. A leading formulation consisting of DLM and a 1:1 mass ratio of HPMC and lecithin stabilizers was successfully scaled up from the 5 mL scale (produced by probe-tip ultrasonication) to the 100 mL scale (produced by high-pressure homogenization). The resulting emulsion was spray dried, and *in vitro* dissolution studies showed significantly enhanced dissolution kinetics compared to crystalline DLM. Further, the spray-dried powder retained its dissolution performance through an accelerated open-vial storage stability protocol (50 °C/75% RH for 4 weeks), and PXRD, DSC, and solid-state NMR confirmed the stability of the formulations against further temperature- and humidity-induced DLM crystallization.

## 2 Materials and Methods

### 2.1 Materials

Delamanid (DLM; 99.9%) was purchased from Gojira Fine Chemicals, LLC (Bedford Heights, OH). Hydroxypropyl methylcellulose (HPMC; Pharmacoat 603), hydroxypropyl methylcellulose phthalate (HPMCP; HP-50), and hydroxypropyl methylcellulose acetate succinate (HPMCAS; AQOAT AS-LF and AS-HF) were generously provided by Shin-Etsu Chemical Co. (Tokyo, Japan). Dichloromethane (DCM; HPLC Plus grade) and Tween 80 were purchased from Sigma-Aldrich (St. Louis, MO). Polycaprolactone diol (PCL; MW 1250 Da) was purchased from Polysciences, Inc. (Warrington, PA). l-α-Lecithin, anisole (99%), and methyl *tert*-butyl ether (MTBE; 99.9%) were purchased from Acros Organics (Geel, Belgium). Tetrahydrofuran (THF; HPLC grade), acetonitrile (HPLC grade), methanol (HPLC grade), chloroform (HPLC grade), ethyl acetate (ACS grade, 99.9%), sodium hydroxide, and trifluoroacetic acid were purchased from Fisher Scientific (Hampton, NH). N-butyl acetate (>99%) was purchased from Alfa Aesar (Ward Hill, MA). HEPES (4-[2-Hydroxyethyl]-1-piperazine ethanesulfonic acid) was purchased from Boehringer Mannheim (Mannheim, Germany). Deionized (DI) water (18.2 MΩ cm) was prepared by a Barnstead Nanopure Diamond ultrapure water system (Thermo Scientific, Waltham, MA).

### 2.2 Methods

#### Preparation of Pre-emulsion Solutions and Mixtures

The solubility of crystalline delamanid was measured in water-immiscible organic solvents suitable for use in the emulsion formulations (SI Figure 1), including dichloromethane (DCM), chloroform, ethyl acetate, anisole, *n*-butyl acetate, and methyl *tert*-butyl ether (MTBE). DCM was selected for use in the emulsion formulations as it displayed the greatest delamanid solubility (6.67% w/w) of all solvents tested, and its high volatility (bp 39.6 °C) enabled near-complete removal.

Prior to emulsification, two stock solutions were prepared: (1) an aqueous solution containing a dissolved water-soluble stabilizer (if desired); and (2) an organic solution containing delamanid and an organic-soluble stabilizer (if desired) dissolved in DCM. HPMC and MC were directly dissolved at 8% w/w in deionized water. Ionizable cellulosic polymers (e.g., HPMCAS and HPMCP) were dissolved in deionized water with stoichiometric addition of NaOH to ionize the succinate and phthalate functional groups, respectively. HPMCAS LF and HPMCP were dissolved at 8% w/w in water, while HPMCAS HF was dissolved at 3% w/w due to solubility limitations. After dissolution of the cellulosic stabilizer, the solution was subsequently diluted with deionized water to achieve the desired concentration of the stabilizer in the aqueous stock solution. Delamanid was dissolved in DCM at 6.67% w/w. Lecithin, if used, was dissolved along with delamanid, such that the solution contained 6.67% w/w delamanid and 5% w/w lecithin in DCM. Prior to emulsification, the aqueous and organic stock solutions were volumetrically mixed (with additional neat DCM or water, as needed) to obtain the desired final concentrations of all components and the desired final ratio of the organic (dispersed) phase to the aqueous (continuous) phase.

#### Preparation of Emulsions by Probe-Tip Ultrasonication

Small batch emulsions were produced by probe-tip ultrasonication to conduct formulation screening. Desired volumes of pre-emulsion aqueous and organic solutions containing dissolved delamanid and stabilizer(s) were added to a 3-dram glass vial to achieve a final volume of 5 mL. A magnetic stir bar was added, and the vial was placed in an ice water bath atop a magnetic stir plate. The pre-emulsion mixture was stirred while sonicated using a VibraCell VC-50 50-W probe-tip ultrasonicator with 1/8” titanium alloy probe (Sonics & Materials, Inc., Danbury, CT). The probe tip was positioned approximately 0.5 cm above the bottom of the sample vial. The experimental setup is shown in SI Figure 2A. Samples were continuously sonicated for 3 min at a frequency of 20 kHz and an intensity of 20%. Particle size was measured by dynamic light scattering immediately after emulsification and periodically over 24 h. Dichloromethane loss during sonication was quantified as a function of sonication time (SI Figures 3 and 4).

#### Preparation of Emulsions by High-Pressure Homogenization

For scale-up evaluation, larger batch (20–200 mL) emulsions were prepared using an EmulsiFlex C5 air-driven high-pressure homogenizer (Avestin Inc., Ottawa, Canada). The homogenizer was immersed in a room temperature water bath (T ∼ 20 °C) and allowed to thermally equilibrate for at least 15 min. The experimental setup is shown in SI Figure 2B. The homogenizer was first primed with water and adjusted to the desired homogenizing pressure (15,000 psi) by regulating the backpressure on the homogenizing valve to approximately 30 psi. Pressure to the feed pump and the homogenizing valve was supplied by a house compressed air feed at approximately 100 psi. After priming, 4 mL of the holdup volume remained in the internal volume of the system. The amount of water in the aqueous phase of each pre-emulsion mixture was reduced by 4 mL to account for this holdup volume.

After priming the system, desired volumes of pre-emulsion aqueous and organic solutions, containing dissolved delamanid and stabilizer(s), were mixed to achieve a final volume of 100 mL. The mixture was manually shaken for 10 s and then added to the homogenizer feed reservoir. The homogenizing valve backpressure was manually regulated during homogenization to maintain the desired homogenizing pressure of 15 000 psi. The homogenized sample was collected after each pass through the system, after which it was readded to the feed reservoir and rehomogenized until the desired number of passes was achieved (typically 3 or 4 passes). Dichloromethane loss during homogenization was measured as a function of batch size and number of passes (SI Figures 3 and 4). Sample temperature was measured for selected batches (SI Figures 5 and 6 and SI Tables 1 and 2). Particle size was measured by dynamic light scattering immediately after emulsification and periodically over 24 h.

#### Particle Size Measurements by Dynamic Light Scattering

The droplet size of the resulting emulsions was characterized by dynamic light scattering (DLS) using a Zetasizer Nano-ZS instrument (Malvern Instruments, Southboro, MA). For measurement, samples were diluted in DCM-saturated water to a final solids concentration of 0.3 mg mL^–1^. Emulsions were characterized by DLS immediately after preparation, as well at “intermediate” (*t* = 3–5 h) and “long” (*t* > 18 h) time points to assess the size stability of the emulsions. Data is reported as intensity weighted particle size distributions with Z-average hydrodynamic diameter. The broadness of the size distribution is characterized by the polydispersity index (PDI); PDI < 0.1 indicates a monodisperse sample, while PDI > 0.7 generally indicates a distribution too broad for accurate DLS measurement. Cumulants analysis of DLS data was performed by the Zetasizer software package in accordance with ISO 13320.

#### Pendant Drop Tensiometry Measurements

The dichloromethane-water interfacial tension in the presence of varying concentrations of stabilizers was measured by using a Drop Shape Analyzer pendant drop tensiometer (Krüss Scientific, Matthews, NC). In brief, a pendant DCM droplet was suspended in aqueous media consisting of DCM-saturated deionized water containing dissolved stabilizer (HPMC, HPMCP, HPMCAS LF, or HPMCAS HF). Measurements were obtained for aqueous solutions containing 0.1%, 0.05%, and 0.01% w/w stabilizer. Unless otherwise noted, droplets were formed using a 20 gauge needle (outer diameter = 0.908 mm). Measurements of the interfacial tension between a dichloromethane droplet and DCM-saturated water were performed at the beginning of each measurement session to ensure agreement with the reported literature value of 28.0 mN/m.^31^

#### Spray Drying of Emulsions

Following high-pressure homogenization (100 mL batch size), HPMC was added as a bulking agent at a 1:1 mass ratio of HPMC to emulsion solids to prevent aggregation during spray drying, in line with previous experience in spray drying nanoparticle formulations.^26,27,32,33^ Samples were spray dried by using a B-290 spray dryer (Büchi Corporation, New Castle, DE) using the following parameters: *T*_inlet_ = 120 °C, *T*_outlet_ = 40–50 °C, solution feed rate ∼7 mL min^–1^. The solution feed was atomized through a 0.7 mm diameter atomizing nozzle with nitrogen used as the atomizing gas (N_2_ flow rate ∼470 L h^–1^). The aspirator was set to a value of 90%, corresponding to a drying gas circulation rate of approximately 35 m^3^ h^–1^. After spray drying, powders underwent an overnight secondary vacuum drying step (*P*_gauge_ = −30 mmHg) at room temperature for further removal of the residual solvent.

#### Residual Dichloromethane Static Headspace Measurements

Residual levels of dichloromethane in spray-dried powders were measured using static headspace gas chromatography with mass spectroscopy detection (SHS-GC/MS). Samples were stored at 4 °C prior to measurement. Approximately 0.1 g of sample was placed in a tared 20 mL headspace vial, and the sample weight was recorded. The vial was immediately sealed and placed in an Agilent Technologies 7697A headspace sampler, where samples were outgassed at 92 °C for 1 h. Headspace gases were analyzed using a Hewlett-Packard 6890 GC with 5973 Mass Selective Detector (Santa Clara, CA). The concentration of DCM was quantified using an external, pure DCM standard. Measurement and analysis were performed by Innovatech Laboratories LLC (Plymouth, MN).

#### Powder X-ray Diffraction

Powder X-ray diffraction (PXRD) measurements were performed using a D8 Advance twin diffractometer (Bruker Corporation, Billerica, MA) equipped with Cu Kα radiation (λ = 1.54 Å) and a LYNXEYE-XE detector. Powder was loaded into a polyimide capillary (inner diameter of 1 mm), which was then mounted on a rotating (60 rpm) capillary stage. Data were collected in the range 2Θ = 3–20° with a step size of 0.025° and count rate of 5 s per step. Analysis was performed using the Bruker DIFFRAC EVA V3.1 software.

#### Differential Scanning Calorimetry

Spray-dried emulsions and ASD powders were thermally characterized by differential scanning calorimetry (DSC). Approximately 10 mg of powder was loaded into an aluminum Tzero DSC pan. Characterization was performed using a TA Instruments Discovery 2500 DSC instrument (New Castle, DE). Each sample was analyzed at a rate of 5 °C min^–1^ over three heating and cooling cycles from 25 to 210 °C. Data analysis was carried out using TA Universal Analysis software.

#### Solid-State ^13^C Nuclear Magnetic Resonance

Solid-state NMR measurements were acquired on an Avance NEO spectrometer (Bruker Corporation, Billerica, MA) with a 7 T superconducting magnet operating at frequencies of 300 and 75 MHz for the ^1^H and ^13^C nuclei, respectively. Approximately 80 mg of sample was packed in 4 mm zirconia rotors and spun to 13 kHz at the magic angle. The ^13^C spectra were acquired with cross-polarization from the ^1^H to ^13^C nuclei using an optimized 1.5 ms ramped 70–100% contact pulse. Recycle delays of 10 s were used to ensure complete relaxation of the ^1^H nuclei, and up to 8k signal transients were coadded for sufficient signal-to-noise. The ^1^H 90° pulse length of 3.7 μs and a Spinal-64 decoupling scheme with a field strength of 69.7 kHz were used during acquisition.

#### *In Vitro* Dissolution Assay and Accelerated Stability Protocol

The dissolution of delamanid from spray-dried powders was measured using an *in vitro* dissolution assay in aqueous media consisting of 150 mM HEPES buffer at pH 7 with 3% w/w Tween 80 based on a previously described protocol.^28^ The solubility of delamanid was measured in various buffers and in commercially available simulated intestinal fluid media (see SI Figure 7 and SI Tables 3 and 4). However, delamanid exhibited limited solubility in the commercial SIF buffers; its solubility limit was near the lower limit of quantification of HPLC-UV, which prevented accurate quantification of the dissolution kinetics. The HEPES-Tween buffer system was chosen, as it offered greater delamanid solubility (23.6 μg mL^–1^). Buffer systems containing Tween surfactant have been shown to be comparable to commercially available SIF buffers.^34−36^

In brief, the spray-dried powder was resuspended in deionized water and then diluted 10-fold into HEPES-Tween buffer prewarmed to 37 °C to obtain a final delamanid concentration of 10–15 μg mL^–1^ and final volume of 10 mL. Vials were held in a 37 °C water bath for the duration of the assay. At *t* = 15, 30, 60, 120, 180, and 360 min, a 1.2 mL aliquot was removed and centrifuged (21 000 *g*, 10 min) to pellet undissolved material. The supernatant (1 mL) was removed, frozen, and lyophilized. Also at *t* = 360 min, an additional 1 mL aliquot of sample was removed and directly frozen and lyophilized without centrifugation to enable determination of the total delamanid concentration in each vial, *C*_0, DLM_. Lyophilization was conducted using a FreeZone Triad shelf lyophilizer (Labconco, Kansas City, MO) with a shelf temperature of −20 °C and vacuum of 0.02 Torr. Deionized water (100 μL) was added to each lyophilized sample to wet the HPMC and salts. Samples were lightly vortexed, and after 15 min, 900 μL of THF was added to solubilize delamanid. Samples were centrifuged (21 000 *g*, 10 min), and the supernatant was analyzed by HPLC to measure the concentration of dissolved delamanid at each time point t, C_t, DLM_. The percent dissolved was calculated as 100 *C*_t, DLM_/*C*_0, DLM_. *In vitro* dissolution measurements were also performed on spray-dried powders subjected to an accelerated 4 week open-vial storage stability protocol (50 °C/75% RH). To maintain the desired temperature and humidity, vials were placed in a sealed chamber containing a NaCl-saturated water solution;^37^ this chamber was placed in a gravity convection oven (VWR International, Radnor, PA) with digital temperature control.

#### Delamanid Quantification by HPLC

Delamanid concentration was quantified by reverse-phase HPLC (Phenomenex Kinetex C18, 100 Å, 150 mm × 4.6 mm, 5 μm particles) using an Agilent 1100 series HPLC (Agilent Technologies, Santa Clara, CA). Samples (10 μL injection volume) were analyzed with an isocratic mobile phase of 60:40 water/acetonitrile with 0.05% v/v trifluoroacetic acid at a flow rate of 1 mL min^–1^ and column temperature of 35 °C. Delamanid was detected using a diode array detector at a UV absorbance of 330 nm (RT = 6.8 min). A linear standard curve was constructed for concentrations between 0.7 and 50 μg mL^–1^ using the integrated area of the UV absorbance peak (SI Figure 8).

## 3 Results and Discussion

### 3.1 Formulation Screening via Probe-Tip Ultrasonication

**Figure 1:**
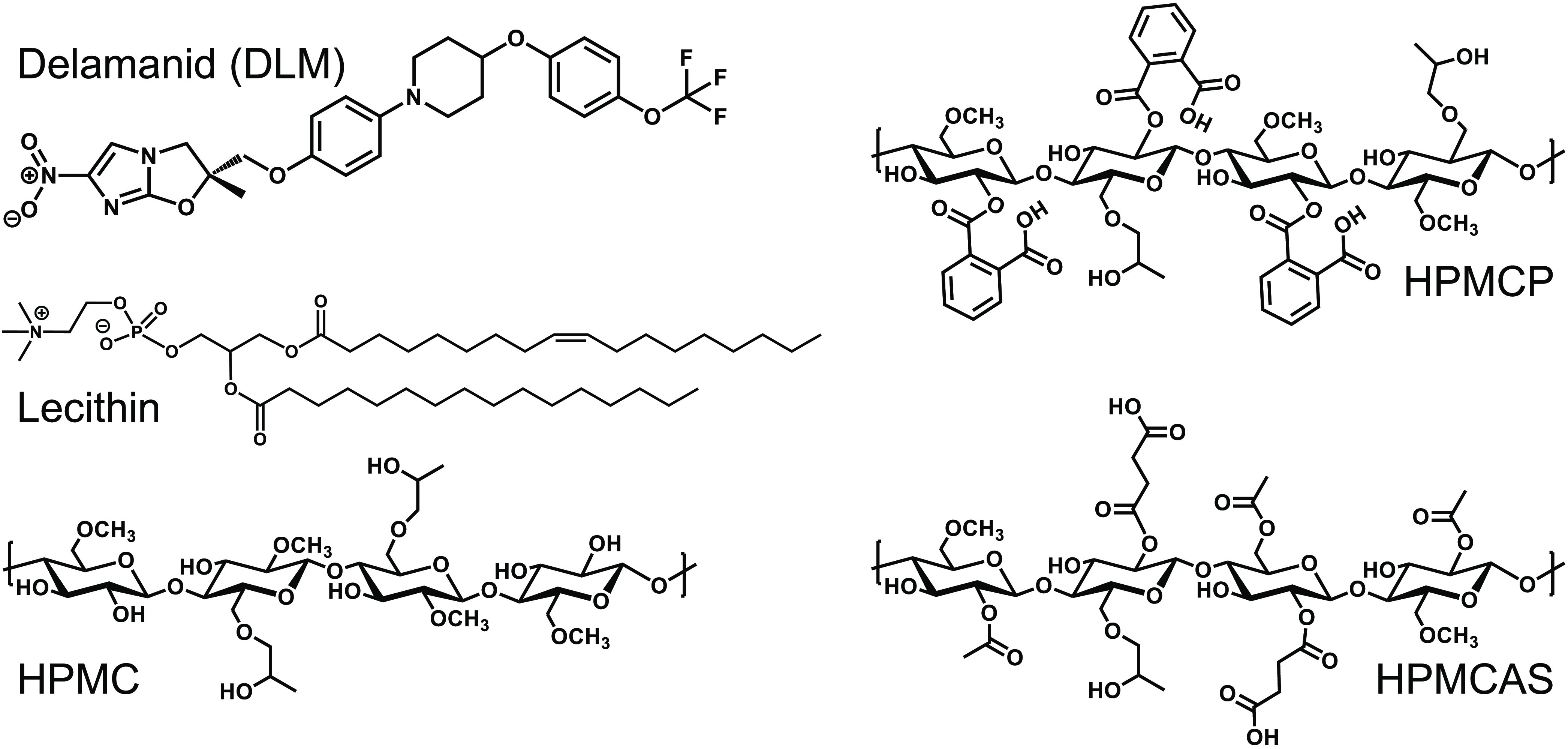
Chemical structures of delamanid (DLM) and emulsion stabilizers investigated: l-α-lecithin, hydroxypropyl methylcellulose (HPMC), hydroxypropyl methylcellulose phthalate (HPMCP), and hydroxypropyl methylcellulose acetate succinate (HPMCAS).

Formulation screening was conducted by preparing small-batch (5 mL) emulsions via probe-tip ultrasonication. During the 3 min sonication time, the average energy input to the emulsion volume was estimated to be on the order of 1 × 10^9^ J m^–3^. However, in reality the energy input is expected to be highly localized to the area immediately surrounding the probe tip. Figure 1 shows the chemical structures of the delamanid (DLM) and the stabilizers investigated in the formulations. Properties of the functionalized cellulosic polymers used are listed in Table 1.

**Table 1:** Properties of Cellulosic Stabilizers Investigated, Provided by Manufacturers^23−25,29,30^

Material	Grade	Ionizable Group	Percent Ionic Substitution (% w/w)	Labeled Viscosity (mPa s)^a^	Molecular Weight (g mol^–1^)
HPMC	Pharmacoat 603	–	–	3	20 000^24^
HPMCP	HP-50	Phthalate	24	55	37 900^25^
HPMCAS LF	AS-LF	Succinate	15	3	18 200^23^
HPMCAS HF	AS-HF	Succinate	7	3	17 400^23^

a 2% w/w aqueous solution at 20 °C.

In all emulsions, the dispersed phase consisted of DLM dissolved in dichloromethane, while the continuous phase was aqueous. Water-soluble stabilizers were dissolved in the continuous phase prior to emulsification. When lecithin was present in the formulation, it was dissolved in the dispersed phase.

Emulsion formulation parameters are listed in Table 2. Key parameters varied during the screening experiments were the total solid concentration, i.e., the sum of the stabilizer and DLM concentrations (in formulations F1–F3), and the dispersed phase fraction (formulations F4–F7). Each formulation was prepared by using HPMC, HPMCP, HPMCAS HF, or HPMCAS LF as a single stabilizer. Later, each formulation was prepared using lecithin as a costabilizer at a 1:1 mass ratio of cellulose to lecithin, keeping the total stabilizer concentration fixed. Tabulated size measurements and time stability data for all formulations are reported in SI Appendix A.

**Table 2:** Composition of the Emulsion Formulations^a^

Formulation	Dispersed Phase (% v/v)	Stabilizer (% w/w)	DLM (% w/w)	DLM loading (%)
F1	15	1.5	1.0	40
F2	15	1.0	0.67	40
F3	15	0.5	0.33	40
F4	20	2.0	1.33	40
F5	25	2.5	1.67	40
F6	30	3.0	2.0	40
F7	40	4.0	2.67	40

a The dispersed phase was dichloromethane and the continuous phase was aqueous. The stabilizer was either a cellulosic polymer or a 1:1 mass ratio of a cellulosic polymer to lecithin as a co-stabilizer. The dispersed phase content of each formulation is reported as a volume fraction, , where *V*_organic_ is the volume of dichloromethane and *V*_total_ is the total volume of the organic and aqueous phases of the emulsion. Stabilizer and DLM concentrations are reported as a weight percent of the total mass of the liquid emulsion formulation. Drug loading is reported as a weight percent of the total mass of solid (nonvolatile) components of the emulsion, , where *m* denotes the mass of the respective components. For formulations containing two stabilizers (i.e., lecithin and a cellulosic stabilizer), the mass ratio of the stabilizers was 1:1.

In formulations F1–F3 the dispersed phase fraction was held at 15% since spray drying using air as the drying gas requires that the organic solvent content of the liquid feed is kept under 20% to avoid ignition of the solvent at high temperature (approximately 120 °C). (It is noted that spray drying mixed organic/aqueous feeds with solvent content above 20% is possible using a closed-loop system with inert atmosphere and solvent and water condensers. In practice, incomplete removal of both solvent and water in the closed loop can result in poor drying efficiency. This is improved at industrial scale.) As a result of the solubility limit of DLM in dichloromethane (6.67% w/w), the maximum DLM concentration attainable in emulsions with 15% dispersed phase was 1% w/w, as reflected in F1. Stabilizer concentrations had been previously investigated in formulations of other drugs and model materials and were maintained as a starting point for studies with DLM. Thus, the starting DLM loading was 40% w/w. In formulations F2 and F3, the concentrations of stabilizer and DLM were decreased while keeping the DLM loading fixed at 40% to investigate the effect of the total solids concentration on the resulting particle size.

Increased dispersed phase content was investigated in formulations F4–F7. Data for formulations F4–F7 are shown in SI Figure 9. Using the stabilizer and DLM concentrations from F1 as a starting point, the dispersed phase fraction was increased incrementally to 40% v/v with fixed DLM loading. The higher concentrations of stabilizer and DLM enabled by the increased dispersed phase content are desirable for scale-up, as they result in increased mass efficiency. While at our laboratory scale we kept DCM concentration below 20% to ensure operation below the lower flammable limit during spray drying, it is possible to employ higher DCM concentrations at industrial scale using inert gas blanketing. Therefore, the data serves as a foundation for further scale-up work.

**Figure 2:**
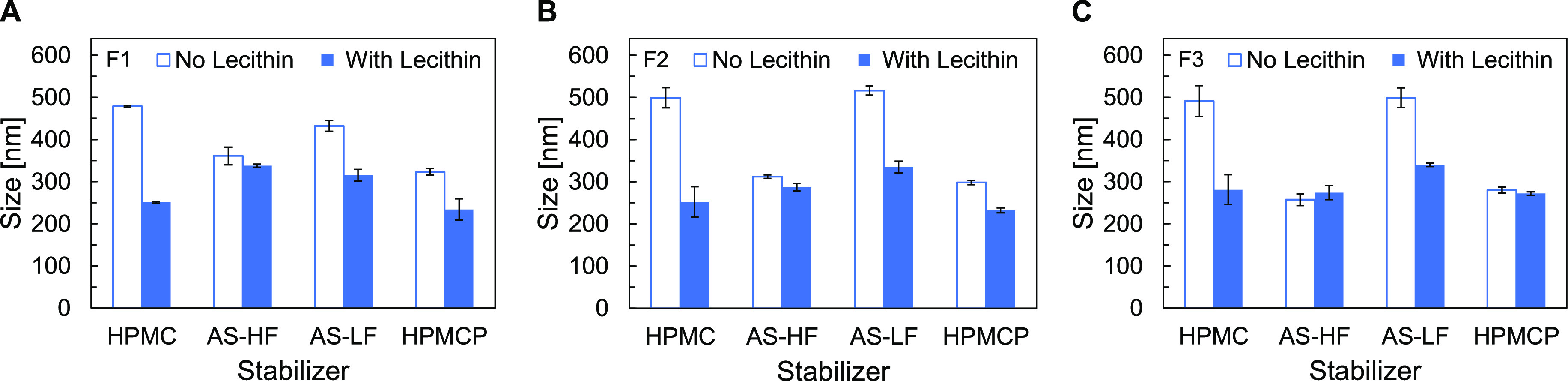
*Z*-average hydrodynamic diameter of emulsion formulations (A) F1, (B) F2, and (C) F3, prepared by probe-tip ultrasonication using HPMC, HPMCAS-HF, HPMCAS-LF, or HPMCP as stabilizer (open bars) or a 1:1 mass ratio of lecithin to each of the cellulosic polymers (filled bars).

Figure 2 shows the particle size measurements of formulations F1–F3 prepared with HPMC, HPMCAS HF, HPMCAS LF, or HPMCP as a single stabilizer and in combination with lecithin at a 1:1 mass ratio.

Formulations produced using a single cellulosic stabilizer displayed average droplet sizes varying from 200 to 500 nm. The electrostatic stabilization imparted by the ionic stabilizers resulted in smaller droplet sizes than formations stabilized by the nonionic HPMC. The exception to this was HPMCAS-LF, which is less substituted with hydrophobic acetate groups than HPMCAS-HF, the other HPMCAS grade tested. The greater hydrophobic functionalization of HPMCAS-HF likely facilitated better stabilization of the dichloromethane-water interface at the droplet surface. While Figure 2 shows size measurements performed soon after emulsification, longer term size stability measurements revealed that all formulations stabilized by only cellulosic stabilizer displayed significant increases in droplet size over a 24 h period. Tabulated size stability data for all formulations are provided in SI Appendix A.

The use of lecithin as a costabilizer in combination with a cellulosic stabilizer resulted in smaller emulsion droplet sizes and improved size stability over time. By themselves, both HPMC and HPMCAS-LF produced emulsion droplets of approximately 400–500 nm across formulations F1–F3. However, with the addition of lecithin as costabilizer, droplet size decreased to 200–300 nm. For example, the average diameter of the HPMC/lecithin-stabilized F2 formulation was 252 ± 36 nm, compared to 499 ± 24 nm for the same F2 formulation stabilized by only HPMC. The HPMC/lecithin-stabilized F2 formulation also displayed better size stability over time; after 24 h its average droplet size was 305 ± 13 nm, while the F2 formulation stabilized by only HPMC was 610 ± 27 nm. Thus, the presence of lecithin as a costabilizer helped prevent emulsion droplet aggregation and coalescence over time.

To investigate the underlying behavior at the surfactant-stabilized emulsion droplet interface, dichloromethane/water pendant drop dynamic interfacial tension (IFT) measurements were performed in the presence of stabilizers used in the emulsion formulations. IFT data was collected as a function of time for each droplet, and the equilibrium interfacial tension γ_∞_ was calculated by linear regression using an infinite-time asymptotic solution to the Ward and Tordai model for modeling adsorption of surfactant to a nondeforming surface:^38,39^
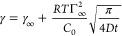
where γ is the dynamic interfacial tension, *R* is the gas constant, *T* is temperature, *C*_0_ is the molar bulk concentration of surfactant, Γ_∞_ is the steady-state molar surface concentration, *D* is the diffusion coefficient, and *t* is time.

**Figure 3:**
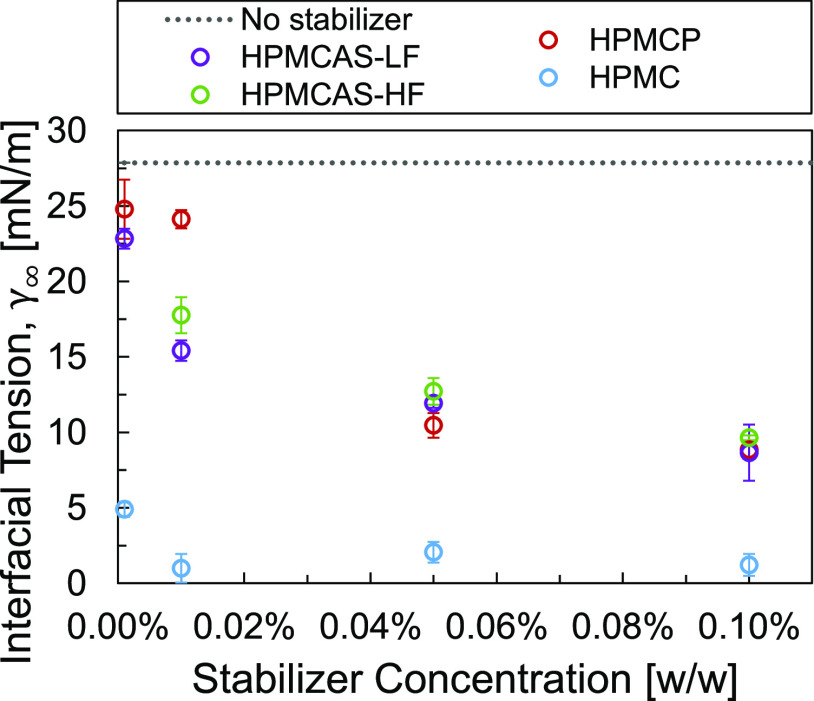
Dichloromethane/water interfacial tension as a function of stabilizer concentration (from 0.001 to 0.1% w/w) for HPMCAS-LF, HPMCAS-HF, HPMCP, and HPMC, measured by pendant drop tensiometry. The neat dichloromethane/water interfacial tension was also measured and is plotted for comparison.

Figure 3 shows the equilibrium IFT values as a function of the stabilizer concentration. Measurements were conducted at dilute stabilizer concentrations; the higher concentrations used in the emulsion formulations were too high to enable formation of a stable pendant droplet. Additionally, the IFT was not measured for systems containing lecithin, as even low concentrations of lecithin decreased IFT substantially, and a stable pendant droplet could not be maintained. As such, the IFT measurements for cellulosic materials were intended to inform formulation design on a relative basis.

However, the IFT measurements alone were not an accurate predictor of the relative droplet sizes displayed by the emulsion formulations. Although HPMC consistently produced the greatest decrease in the IFT across all concentrations of the stabilizers investigated, emulsion formulations stabilized by HPMC consistently displayed the largest average droplet sizes. This suggests that although HPMC makes the formation of additional interfacial areas energetically favorable, it is not effective at preventing droplet aggregation and coalescence due to the presence of only weak steric stabilization. Additionally, the IFT measurements do not capture the differences in behavior between the ionic stabilizers HPMCAS-LF, HPMCAS-HF, and HPMCP. These three stabilizers displayed similar IFT behavior over the concentration range studied; however, while HPMCAS-HF and HPMCP produced similar emulsion droplet sizes, the less hydrophobically substituted HPMCAS-LF produced larger droplet sizes, as shown in Figure 2 and discussed in the preceding paragraphs. Lastly, in the emulsion formulations, delamanid may also act as a cosurfactant owing to the presence of its zwitterionic nitro group. However, the size data in Figure 2 suggests that droplet size is highly dependent on the external stabilizer(s) introduced to the system.

The HPMC/lecithin-stabilized formulations F1 and F2 were selected for further investigation and scale-up. These formulations produced sub-300 nm emulsion droplets that were size stable over a period of 24 h. While other lecithin/cellulose stabilizer combinations yielded similar initial droplet sizes, HPMC/lecithin-stabilized formulations consistently displayed the best size stability over time (i.e., the least increase in average droplet size over 24 h). (Tabulated size stability data are provided in SI Appendix A.) As an added benefit, the nonionic HPMC can be directly dissolved in aqueous media without the need for neutralization of ionizable substituents. F1 and F2 were investigated in tandem because F2 contains delamanid at a concentration slightly below its solubility limit in the dichloromethane dispersed phase. When scaling up the process using high-pressure homogenization, some amount of solvent loss was expected as a result of the high energy input imparted to the emulsion. In the event of solvent loss, formulation F2 reduces the risk of premature drug precipitation during emulsification as compared to F1, which contains delamanid in the dispersed phase at a concentration near its solubility limit.

### 3.2 Emulsion Scale up via High-Pressure Homogenization

While ultrasonication is a convenient method for preparing emulsions at a small scale, it is limited by the rapid dissipation of ultrasonic energy in liquid media. As a result, ultrasonication provides a high-intensity energy input only to a localized area immediately surrounding the probe tip, making it difficult to apply energy uniformly to larger sample volumes. In contrast, high-pressure homogenization provides uniform energy input to large sample volumes by forcing the liquid feed through a small orifice at a high pressure. The resulting extensional shear flow enables a uniform emulsion droplet breakup. The energy input from a high-pressure valve homogenizer may be estimated from the homogenizing pressure.^40^ Here, we estimate the energy input to be on the order of 1 × 10^8^ J m^–3^. This is similar to the average energy input of 10^9^ J m^–3^ estimated for the probe-tip ultrasonicator used for the small batch samples, although during sonication, the local energy input varies greatly throughout the sample volume.

The lead HPMC/lecithin-stabilized formulations F1 and F2 were investigated for scale-up via high-pressure homogenization using an air-driven Avestin EmulsiFlex C5 homogenizer. This lab-scale homogenizer has a throughput of 1–5 L/h and can accommodate batch sizes from approximately 20 mL to several liters. The process is continuous, and the liquid feed can be recycled to undergo multiple homogenization passes until the desired droplet size is achieved.

Initially, homogenization process parameters on the EmulsiFlex C5 were investigated using formulation F1 but substituting polycaprolactone (PCL) for DLM as the core material. Using a homogenization pressure of approximately 15 000 psi, emulsion droplet diameter was investigated as a function of homogenization passes and was compared to droplet diameters produced by ultrasonication (SI Figure 10). This initial investigation showed that high-pressure homogenization could produce emulsion droplets with size distribution and size stability nearly identical to that produced by ultrasonication. Importantly, this demonstrated that small-scale screening experiments using ultrasonication could predictably be translated to large-scale homogenization processing.

**Figure 4:**
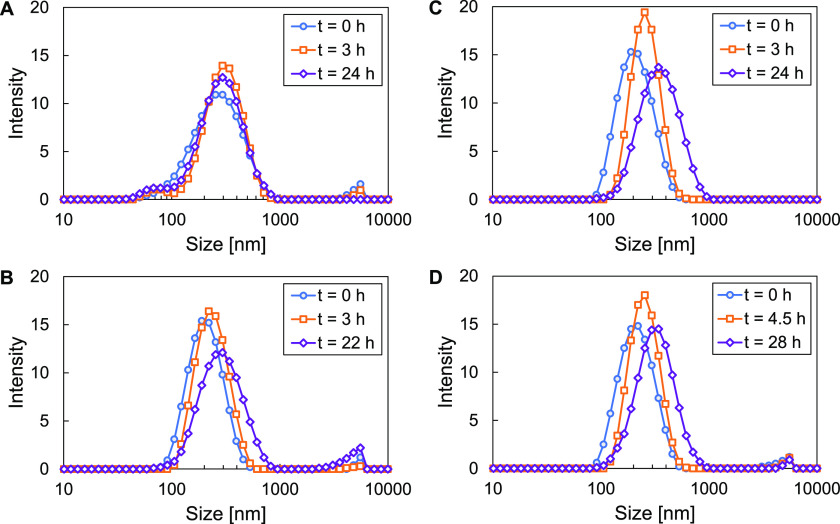
Particle size distributions of F1 DLM/HPMC-lecithin emulsion formulation prepared by (A) probe-tip ultrasonication and (B) high-pressure homogenization and particle size distributions of F2 DLM/HPMC-lecithin emulsion formulation prepared by (C) probe-tip ultrasonication and (D) high-pressure homogenization.

Following the initial homogenization process parameter determination, DLM-containing emulsion formulations F1 and F2 were prepared on a 100 mL scale via high-pressure homogenization. Figure 4 shows the particle size distributions over time of the F1 and F2 formulations prepared by probe-tip ultrasonication (5 mL scale) and high-pressure homogenization (100 mL scale). Table 3 contains tabulated size and PDI values. For both formulations, ultrasonication and homogenization produced droplets with similar initial size distribution and similar behavior over 24 h of storage time. This size stability was sufficient to enable subsequent spray drying. However, DLM-containing formulations displayed more significant size increase over time than the control formulations containing PCL as the core (shown in SI Figure 10). This ripening behavior in the DLM-containing formulations is likely driven by gradual loss of solvent from the core of the emulsion droplets. Initially, DLM is completely solubilized in the liquid dichloromethane core of the emulsion droplet. However, as the volatile solvent gradually partitions into the aqueous and vapor phases, DLM begins to precipitate and form a solid core. As discussed previously, the trifluoromethyl and nitro functional groups of the DLM likely cause incompatibility at the solid DLM—stabilizer interface, leading to the shedding of the stabilizer and a gradual increase in droplet size. In contrast, the PCL core used in the control studies is compatible with the stabilizers and does not display significant size increase over 24 h of storage.

**Table 3:** *Z*-Average Hydrodynamic Diameter and PDI over Time for F1 and F2 Emulsion Formulations Containing DLM and Stabilized by a 1:1 Ratio of HPMC to Lecithin Prepared by Probe-Tip Ultrasonication and High-Pressure Homogenization

		*t* ∼ 0 h		*t* ∼ 3 h		*t* ∼ 24 h	
Formulation	Preparation method	Size (nm)	PDI	Size (nm)	PDI	Size (nm)	PDI
F1	Ultrasonication	251 ± 2	0.29 ± 0.01	276 ± 6	0.31 ± 0.01	292 ± 6	0.37 ± 0.02
DLM/HPMC-lecithin							
	Homogenization	222 ± 3	0.30 ± 0.03	233 ± 1	0.17 ± 0.03	293 ± 5	0.29 ± 0.02
F2	Ultrasonication	196 ± 3	0.12 ± 0.01	248 ± 4	0.05 ± 0.03	313 ± 3	0.16 ± 0.01
DLM/HPMC-lecithin							
	Homogenization	215 ± 1	0.22 ± 0.04	259 ± 5	0.32 ± 0.03	326 ± 7	0.28 ± 0.02

The stability behavior of the DLM-containing droplets illustrates two important points. First, the emulsion formulation strategy is advantageous because maintaining DLM in a dissolved state in a liquid core allows the formation of stabilized emulsion droplets. In contrast, approaches to encapsulate DLM through the formation of a solid core suffer from instability, as the incompatibility between DLM and the stabilizer prevents stable surface attachment of the stabilizer to the core. Second, since gradual loss of solvent from the liquid core leads to droplet size increase and eventual destabilization, it is important that solvent be removed rapidly when the emulsion is dried to form a powder. In this way, the integrity of the core–shell emulsion droplets will be maintained. For this reason, spray drying was selected as the drying method to obtain a bulk powder from the liquid emulsion formulations as the liquid phase is removed on the order of 1 s during the drying process.

### 3.3 Spray Drying and Characterization by PXRD and DSC

For global health applications, solid powder drug products are desirable to avoid the prohibitively high costs associated with transporting liquid formulations. Additionally, it is important for the powder product to be stable in high temperature and high humidity environments without the need for expensive barrier packaging. To this end, we investigated spray drying of the emulsion formulations.

Emulsion formulations F1 and F2 (containing DLM dissolved in a dispersed dichloromethane phase stabilized by a 1:1 mass ratio of HPMC to lecithin stabilizer) were rapidly dried by spray drying to produce a solid powder. The spray drying served two purposes: (1) to dry the emulsion droplets rapidly (on the order of 1 s), avoiding the destabilization that would likely be caused with slower drying techniques, and (2) to remove dichloromethane to an acceptable level in the final powder, in accordance with FDA guidelines. Differential scanning calorimetry (DSC), powder X-ray diffraction (PXRD), solid-state NMR, and *in vitro* dissolution kinetic measurements were performed on the resulting powders. The residual level of dichloromethane was also assayed. The powders were then subjected to accelerated stability testing (open vial, 50 °C/75% RH) for 4 weeks, after which the DSC, PXRD, NMR, and *in vitro* dissolution kinetics measurements were performed again to assess the stability of the drug product.

Immediately prior to spray drying, HPMC was added to the emulsion as a bulking agent to reduce the extent of particle fusion and aggregation during the drying process. Previous experience spray drying nanoparticle formulations informed the ratio of bulking agent to emulsion droplets used (here, a 1:1 mass ratio).^26,27,32,33^ Spray drying afforded a white to off-white powder, which was easily recoverable from the collection vessel of the spray dryer. SEM images of powders are shown in SI Figure 11. Static headspace gas chromatography/mass spectrometry measurements of the spray-dried powder showed residual levels of dichloromethane in the F1 and F2 powders were 52.0 and 44.3 ppm, respectively. After subsequent room temperature overnight vacuum drying, residual dichloromethane levels in the powders decreased to 0.5 and 0.6 ppm, respectively. The US FDA classifies dichloromethane as a Class 2 solvent and recommends a maximum concentration of 600 ppm in any drug product.^41^ Residual solvent levels both after spray drying and after secondary drying comply with FDA guidance. Thus, the high volatility of dichloromethane enabled near-complete solvent removal during spray drying.

**Figure 5:**
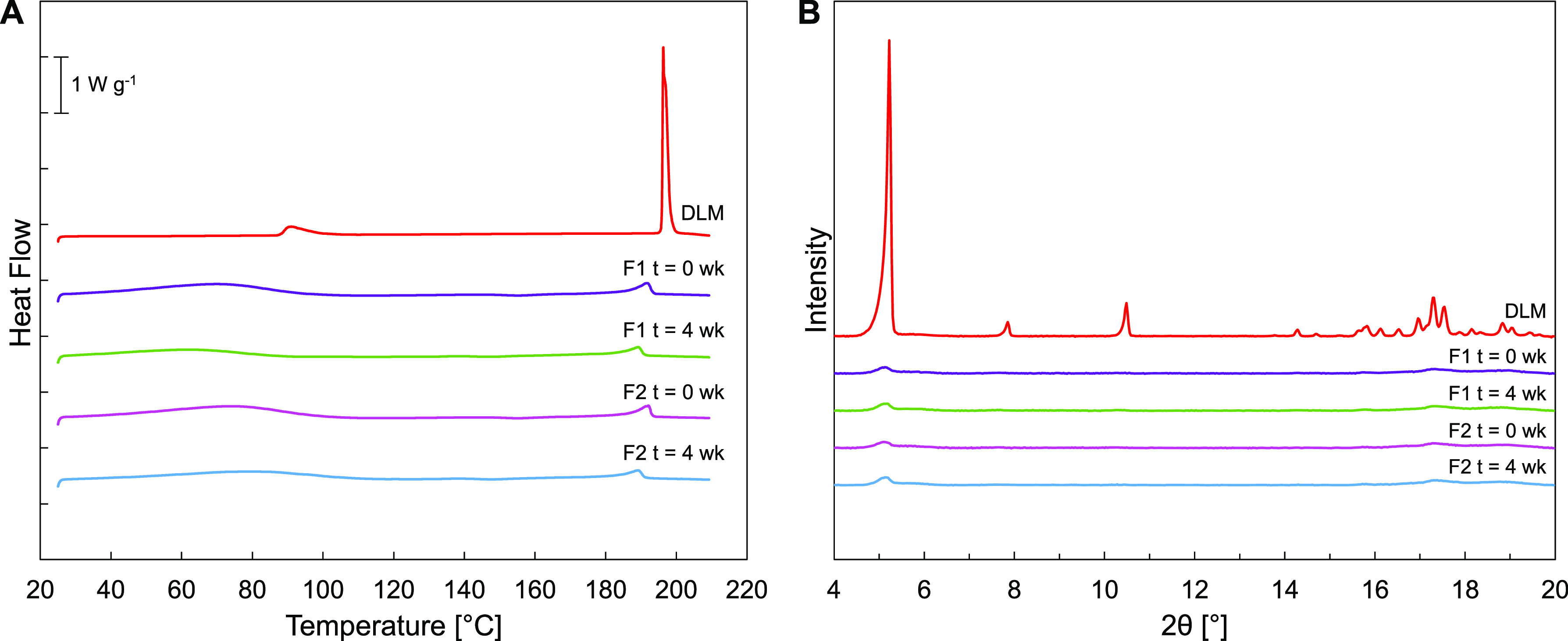
(A) Differential scanning calorimetry (DSC) thermograms and (B) powder X-ray diffraction (PXRD) spectra for F1 and F2 DLM/HPMC-lecithin emulsions at initial time points and after 4 weeks of open-vial storage at 50 °C/75% RH. The thermogram and spectrum of the as-received pure DLM (crystalline) are included for reference.

DLM crystallinity before and after accelerated stability testing was characterized first by DSC and PXRD. Initial DSC studies indicated that DLM has a high tendency to recrystallize from its melt state at cooling rates up to 20 K min^–1^ (SI Figure 12). Figure 5A shows DSC thermograms of pure DLM and of spray-dried emulsion formulations F1 and F2 before and after the 4-week accelerated stability testing. The melting endotherm of DLM was observed at 196 °C in the pure material. In the emulsions F1 and F2, this endotherm was observed at a slightly depressed temperature and with reduced magnitude, suggesting that the materials were partially crystalline. This observed endotherm remained relatively unchanged between the initial state and after the 4-week accelerated stability test. A broad water loss peak was also observed between 60 and 100 °C. Figure 5B shows the PXRD spectra of the spray-dried powders and pure DLM. While the pure material displayed a sharp primary Bragg peak at 5° 2θ, this peak was still present but significantly suppressed in emulsion formulations F1 and F2, indicating some residual crystallinity in the spray-dried emulsion powders. This 5° 2θ peak remained approximately constant even after 4 weeks of accelerated open-vial stability testing at 50 °C/75% RH.

### 3.4 Solid-State NMR Measurements

**Figure 6:**
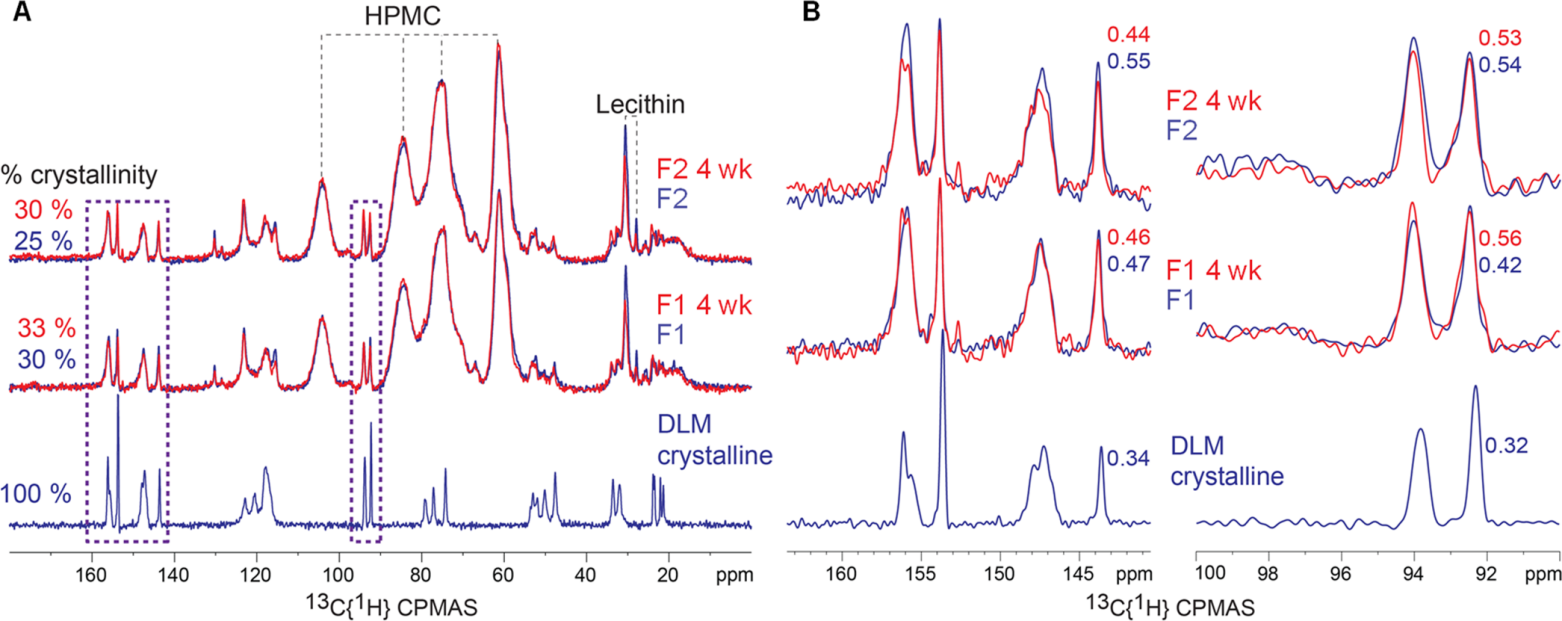
(A) ^13^C solid-state NMR of pure crystalline DLM and spray-dried F1 and F2 DLM/HPMC-lecithin emulsions. The estimated (lower limit) percent crystallinity of DLM in the different formulations is annotated on the left (calculated with respect to the mass of DLM in each formulation). The 4-week (open vial, 50 °C/75% RH) formulations are plotted in red and overlaid on the respective *t* = 0 formulations (blue). The dashed rectangles highlight the specific regions where the DLM signals are nominally well resolved and are not overlapped by signals of the excipients. (B) ^13^C solid-state NMR spectra of the various formulations zoomed in on selected regions highlighting the DLM-only peaks. The change in peak line widths of selected DLM peaks at 143.63 and 92.32 ppm are highlighted next to the peaks.

Solid-state NMR measurements were conducted to provide a quantitative estimate of crystallinity in the spray-dried powders. The ^13^C solid-state NMR of the pure crystalline DLM and the F1 and F2 formulations are presented in Figure 6A. The crystalline DLM yielded well-resolved, narrow signals (Lorentzian line shapes), which reflect the well-defined molecular conformations of the molecule in the crystalline state (for peak assignments see SI Figure 13). Note that for the crystalline DLM, peak multiplets were observed. For example, the spectrum shows four resolved signals of the −CH_3_ site alone, which indicates at least four distinct conformations in the asymmetric unit cell. The spectra for the F1 and F2 formulations have the expected additional signals of the HPMC and the lecithin, which are highlighted by the dashed lines in Figure 6A. The HPMC is completely amorphous, and as a result the signals originating from the HPMC are significantly broadened (Gaussian lineshapes), reflecting the distribution of molecular conformations found in glassy or amorphous systems. The ^13^C NMR signal of the lecithin also yielded relatively narrow peaks; however, for lecithin, the line narrowing does not arise from an ordered crystalline structure but instead due to residual molecular motions as a result of its low glass transition temperature.

Typically, a reduction in crystallinity results in peak broadening and, in favorable systems, a shift in peak positions as seen in our previous work.^32,42^ In the present case, the spray-dried DLM emulsions exhibited a significant broadening of the DLM signals, although no peak shift was observed. Selected DLM signals highlighted by the dashed squares in Figure 6A are plotted in Figure 6B, where the peak broadening is evident. Figure 6B shows the changes in the fwhm (Full Width at Half Maximum) of two DLM peaks at 92.32 and 143.63 ppm. The crystalline DLM, which has large crystals on the micron scale, yielded the narrowest peaks with line widths of 0.32 and 0.34 ppm, respectively. In comparison, the line widths of the F1 and F2 formulations were broadened with line widths of ∼0.44 ppm and ∼0.55 ppm. Following exposure to elevated temperature and humidity during accelerated stability testing, an observed narrowing of the lineshapes could suggest increased crystallinity. However, within the limits of the experimental uncertainty, there was no significant change in the line widths of the of the *t* = 0 and the *t* = 4 week formulations, as highlighted in the virtual overlap of the signals from all the components in the *t* = 0 and *t* = 4 week formulations in Figure 6A. This stability against further crystallization is desirable behavior and demonstrates the exceptional stability of the formulations.

The crystallinity of the DLM was evaluated by taking a scaled difference between the ^13^C NMR spectrum of the formulation and that of the crystalline DLM, which yielded the signal of the disordered DLM fraction. The percent crystallinity was calculated as the difference in the integral of the 92 and 94 ppm peaks before and after subtraction. Accordingly, we estimate that in the F1 and F2 formulations at least 30% and 25% of the DLM is crystalline, respectively. Here it is important to note that in principle there may be a higher fraction of the DLM in the crystalline phase; however, the crystalline size must be much smaller than ∼100 nm, in which case the NMR line widths would revert to that observed of the pure DLM. After 4 weeks at 50 °C/75% RH, the ^13^C NMR peak widths of the DLM in formulations F1 and F2 displayed minimal change, and the crystallinity of the DLM in each formulation was calculated to be 33% and 30%, respectively. This is within the experimental error, and thus we conclude that no significant increase in crystallinity occurred during the accelerated stability study.

For comparison, an amorphous solid dispersion (ASD) formulation containing 20% DLM and 80% HPMCP (HPMCP is used in the commercial formulation Deltyba) was also prepared to investigate its behavior during the same accelerated stability testing protocol. In the case of this ASD, changes in the NMR spectra were clearly evident after 4 weeks (see SI Figure 13 and SI Figure 14). Initially the DLM in the ASD was completely amorphous, as evidenced by broadening of the DLM peaks by over an order of magnitude to >4 ppm. However, 4 weeks of open-vial 50 °C/75% RH storage induced substantial recrystallization of DLM, resulting in the formation of a crystalline phase with significantly narrower line widths of 0.7 ppm -0.8 ppm. Thus, the NMR results indicate that while the F1 and F2 formulations resulted in a relatively higher degree of initial crystallinity of the DLM as compared to the ASD, the spray-dried emulsions were more stable to the effects of elevated temperature and humidity.

### 3.5 *In Vitro* Dissolution Measurements

**Figure 7:**
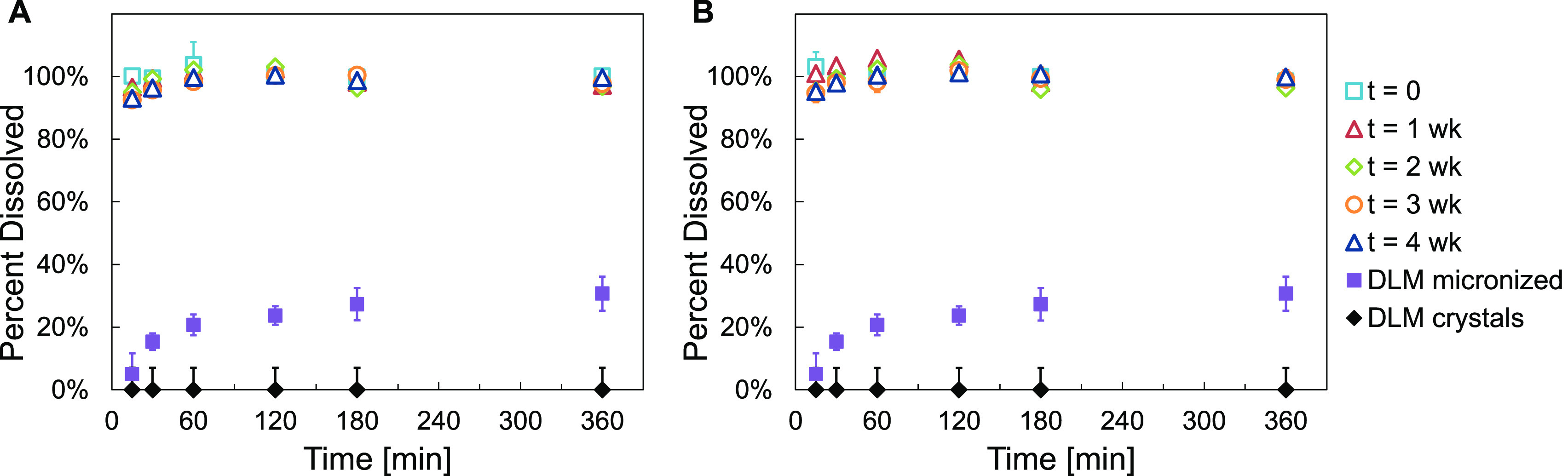
*In vitro* dissolution profiles for spray-dried emulsion formulations (A) F1 and (B) F2 at *t* = 0 and after accelerated storage stability testing (open vial, 50 °C/75% RH) for *t* = 1, 2, 3, and 4 weeks. The emulsion formulations contained DLM and a 1:1 mass ratio of HPMC to lecithin as the stabilizer, with additional HPMC added as a bulking agent prior to spray drying (1:1 mass ratio of HPMC to total nanoparticle mass). The dissolution profiles of the as-received crystalline DLM (∼100 μm) and of micronized DLM (97% crystalline, ∼ 5 μm) are included for comparison. All data points for the DLM crystals were below the HPLC limit of detection (0.7 μg mL^–1^), which is reflected in the error bars.

*In vitro* dissolution measurements were performed on the spray-dried powders before and at times of 1, 2, 3, and 4 weeks during the accelerated stability testing protocol to determine the effect of elevated temperature and humidity on the dissolution performance of the drug product. Dissolution measurements were also performed on the as-received crystalline DLM powder for comparison. Figure 7 shows the *in vitro* dissolution profiles for both F1 and F2 spray-dried emulsion formulations after spray drying (*t* = 0) and at 1 week intervals during the accelerated stability testing protocol.

The crystalline DLM displayed negligible dissolution over the 6 h duration of the assay; all samples were below the HPLC limit of detection of 0.7 μg mL^–1^. This poor dissolution was likely due to the poor aqueous solubility of the crystalline form and the low specific surface area of the ∼100 μm crystals (SI Figure 15A). In contrast, both emulsion formulations F1 and F2 displayed greatly enhanced dissolution kinetics, exhibiting rapid and complete release within 15 min for the t = 0 powders and within 60 min after 4 weeks of accelerated stability testing. This rapid dissolution was likely driven in part by the increased specific surface area of the ∼200 nm emulsion particles compared to the micron-sized crystals.^43^ As indicated by the NMR measurements, the crystallinity of the emulsion powders did not substantially increase after 4 weeks at 50 °C/75% RH, and therefore, the dissolution performance remained nearly the same.

As a comparison, micronized DLM was prepared by spray drying directly from a solution without added excipients to produce ∼5 μm particles (SI Figure 15B). Although it was anticipated that spray drying from solution would render the DLM amorphous, the resulting material was in fact 97% crystalline. This was calculated by comparing the enthalpies of the melting endotherm of the spray-dried DLM to that of the as-received crystalline DLM, both measured by differential scanning calorimetry, as shown in SI Figure 16. However, the dissolution kinetics of this micronized DLM were still limited; only 30% dissolution was achieved over 6 h. This suggests that not only reduced particle size, but also the increased thermodynamic solubility of the partially amorphous DLM in the emulsion powders, are factors contributing to the enhanced dissolution kinetics.

Additionally, the amorphous solid dispersion (ASD) formulation containing 20% DLM and 80% HPMCP initially displayed rapid and complete dissolution kinetics similar to those displayed by the emulsion formulations. However, the dissolution performance was significantly diminished after the 4-week 50 °C/75% RH open-vial stability study (SI Figure 17). This again agrees with the NMR results, which showed that substantial DLM crystallization occurred during the accelerated stability testing, leading to a diminished dissolution performance. This suggests that the phase-separated core–shell geometry of the spray-dried emulsion was more effective than the HPMCP polymer matrix at limiting drug diffusion and crystallization when it was exposed to harsh storage conditions.

Further emulsion formulation development has explored emulsions with higher DLM loadings as well as reduced levels of HPMC bulking agent employed during spray drying. One formulation of interest contains 55% DLM loading in the emulsion droplets and a final DLM loading of 43% DLM in the spray-dried powder. This powder also exhibited rapid and near-complete dissolution of DLM in the *in vitro* assay and stability under the accelerated stability testing protocol (SI Figure 18). Note that the focus of the formulation development described here was a final DLM powder loading of 20% w/w, which was near the maximum loading achieved for stable DLM amorphous solid dispersions.^13^ The ability to produce stable 43% loaded DLM powders by emulsion processing is thus a significant advance that has the potential to decrease dosing mass requirements for DLM while maintaining storage stability.

## 4 Conclusions

Emulsification followed by rapid solidification via spray drying was used as a formulation technique to nanoencapsulate delamanid, a hydrophobic small-molecule therapeutic indicated for treatment of drug-resistant tuberculosis. The trifluoromethyl and nitro functional groups of DLM resulted in poor stabilizer attachment during the formation of a solid DLM core via precipitation- and self-assembly driven nanoencapsulation methods. During emulsification, DLM remained dissolved in the dispersed dichloromethane phase, thereby avoiding the challenges of poor stabilizer attachment. Formulation development demonstrated that naturally derived cellulosic stabilizers and lecithin could produce nanosized DLM-loaded emulsion droplets.

A 1:1 mass ratio of lecithin and HPMC was effective at producing size-stable ∼250 nm emulsion droplets with a 40% DLM loading. The combination of the low molecular weight lecithin, which enabled rapid emulsion stabilization by fast diffusion to the droplet interface, and the polymeric HPMC, which provided additional steric stabilization, enabled the formation of stable, nanosize DLM emulsions. The formulation was successfully scaled up from the 5 mL scale (produced by probe-tip ultrasonication) to 100 mL (produced by high-pressure homogenization). The resulting emulsions were spray dried, and *in vitro* dissolution studies showed significantly enhanced dissolution kinetics compared to crystalline DLM and micronized crystalline DLM. The spray-dried powders retained their dissolution performance through an accelerated open vial storage stability protocol (50 °C/75% RH for 4 weeks). Solid-state NMR measurements showed that these spray-dried emulsion powders were initially partially crystalline, but the percent crystallinity did not increase further during stability testing. A comparative DLM-HPMCP amorphous solid dispersion formulation exhibited significant DLM recrystallization over the same stability testing protocol despite being initially amorphous. Future *in vivo* PK studies could confirm the increase in oral bioavailability and stability predicted by the *in vitro* results.

## Supporting Information Available

DLM solubility data; pictures of experimental setup; HPLC calibration curves; solvent loss and temperature measurements; tabulated emulsion size data; tabulated interfacial tension data; SEM images of formulations; DSC data for pure DLM; NMR data and *in vitro* release for ASD formulation (PDF)

## References

[ref1] Sakula A. Robert Koch: Centenary of the Discovery of the Tubercle Bacillus, 1882. Thorax 1982, 37 (4), 246–251. 10.1136/thx.37.4.246. 6180494 PMC459292

[ref2] Global Tuberculosis Report 2022; World Health Organization: Geneva, Switzerland, 2022.

[ref3] Global Tuberculosis Report 2019; World Health Organization: Geneva, Switzerland, 2019.

[ref4] WHO Consolidated Guidelines on Tuberculosis. Module 4, Treatment: Drug-Susceptible Tuberculosis Treatment; World Health Organization: Geneva, Switzerland, 2022. 35727905

[ref5] WHO Consolidated Guidelines on Tuberculosis. Module 4, Treatment: Drug-Resistant Tuberculosis Treatment; World Health Organization: Geneva, Switzerland, 2022. 36630546

[ref6] Deltyba European Public Assessment Report; , 2013.

[ref7] Pretomanid European Public Assessment Report; European Medicines Agency, 2020.

[ref8] European Medicines Agency. Deltyba. https://www.ema.europa.eu/en/medicines/human/EPAR/deltyba (accessed 2023-02–01).

[ref9] European Medicines Agency. Dovprela (previously Pretomanid FGK). https://www.ema.europa.eu/en/medicines/human/EPAR/dovprela-previously-pretomanid-fgk (accessed 2023-02–01).

[ref10] Piccaro G.; Poce G.; Biava M.; Giannoni F.; Fattorini L. Activity of Lipophilic and Hydrophilic Drugs against Dormant and Replicating Mycobacterium Tuberculosis. J. Antibiot. (Tokyo) 2015, 68 (11), 711–714. 10.1038/ja.2015.52. 25944535

[ref11] Li N.; Cape J. L.; Mankani B. R.; Zemlyanov D. Y.; Shepard K. B.; Morgen M. M.; Taylor L. S. Water-Induced Phase Separation of Spray-Dried Amorphous Solid Dispersions. Mol. Pharmaceutics 2020, 17 (10), 4004–4017. 10.1021/acs.molpharmaceut.0c00798. PMC7539301 32931293

[ref12] Purohit H. S.; Taylor L. S. Phase Separation Kinetics in Amorphous Solid Dispersions Upon Exposure to Water. Mol. Pharmaceutics 2015, 12 (5), 1623–1635. 10.1021/acs.molpharmaceut.5b00041. 25853391

[ref13] Duong T. V.; Nguyen H. T.; Taylor L. S. Combining Enabling Formulation Strategies to Generate Supersaturated Solutions of Delamanid: In Situ Salt Formation during Amorphous Solid Dispersion Fabrication for More Robust Release Profiles. Eur. J. Pharm. Biopharm. 2022, 174, 131–143. 10.1016/j.ejpb.2022.04.002. 35413402 PMC9084191

[ref14] Johnson B. K.; Prud’homme R. K. Flash NanoPrecipitation of Organic Actives and Block Copolymers Using a Confined Impinging Jets Mixer. Aust. J. Chem. 2003, 56 (10), 1021 10.1071/CH03115.

[ref15] Cametti M.; Crousse B.; Metrangolo P.; Milani R.; Resnati G. The Fluorous Effect in Biomolecular Applications. Chem. Soc. Rev. 2012, 41 (1), 31–42. 10.1039/C1CS15084G. 21691620

[ref16] Pigliacelli C.; Maiolo D.; Nonappa; Haataja J. S.; Amenitsch H.; Michelet C.; Sánchez Moreno P.; Tirotta I.; Metrangolo P.; Baldelli Bombelli F. Efficient Encapsulation of Fluorinated Drugs in the Confined Space of Water-Dispersible Fluorous Supraparticles. Angew. Chem., Int. Ed. 2017, 56 (51), 16186–16190. 10.1002/anie.201710230. 29105938

[ref17] Wilson C. J.; Wilson D. A.; Feiring A. E.; Percec V. Disassembly via an Environmentally Friendly and Efficient Fluorous Phase Constructed with Dendritic Architectures. J. Polym. Sci. Part Polym. Chem. 2010, 48 (11), 2498–2508. 10.1002/pola.24046.

[ref18] Floury J.; Desrumaux A.; Lardières J. Effect of High-Pressure Homogenization on Droplet Size Distributions and Rheological Properties of Model Oil-in-Water Emulsions. Innov. Food Sci. Emerg. Technol. 2000, 1 (2), 127–134. 10.1016/S1466-8564(00)00012-6.

[ref19] Qian C.; McClements D. J. Formation of Nanoemulsions Stabilized by Model Food-Grade Emulsifiers Using High-Pressure Homogenization: Factors Affecting Particle Size. Food Hydrocoll. 2011, 25 (5), 1000–1008. 10.1016/j.foodhyd.2010.09.017.

[ref20] Alliod O.; Almouazen E.; Nemer G.; Fessi H.; Charcosset C. Comparison of Three Processes for Parenteral Nanoemulsion Production: Ultrasounds, Microfluidizer, and Premix Membrane Emulsification. J. Pharm. Sci. 2019, 108 (8), 2708–2717. 10.1016/j.xphs.2019.03.026. 30946842

[ref21] Peshkovsky A. S.; Bystryak S. Continuous-Flow Production of a Pharmaceutical Nanoemulsion by High-Amplitude Ultrasound: Process Scale-Up. Chem. Eng. Process. Process Intensif. 2014, 82, 132–136. 10.1016/j.cep.2014.05.007.

[ref22] Anton N.; Benoit J.-P.; Saulnier P. Design and Production of Nanoparticles Formulated from Nano-Emulsion Templates—A Review. J. Controlled Release 2008, 128 (3), 185–199. 10.1016/j.jconrel.2008.02.007. 18374443

[ref23] Fukasawa M.; Obara S. Molecular Weight Determination of Hypromellose Acetate Succinate (HPMCAS) Using Size Exclusion Chromatography with a Multi-Angle Laser Light Scattering Detector. Chem. Pharm. Bull. (Tokyo) 2004, 52 (11), 1391–1393. 10.1248/cpb.52.1391. 15516773

[ref24] Shin Etsu Pharmacoat Product Catalog; Shin-Etsu Chemical Co., Ltd., 2018.

[ref25] Fukasawa M.; Obara S. Molecular Weight Determination of Hypromellose Phthalate (HPMCP) Using Size Exclusion Chromatography with a Multi-Angle Laser Light Scattering Detector. Chem. Pharm. Bull. (Tokyo) 2003, 51 (11), 1304–1306. 10.1248/cpb.51.1304. 14600378

[ref26] Zhang Y.; Feng J.; McManus S. A.; Lu H. D.; Ristroph K. D.; Cho E. J.; Dobrijevic E. L.; Chan H.-K.; Prud’homme R. K. Design and Solidification of Fast-Releasing Clofazimine Nanoparticles for Treatment of Cryptosporidiosis. Mol. Pharmaceutics 2017, 14 (10), 3480–3488. 10.1021/acs.molpharmaceut.7b00521. PMC5627342 28929769

[ref27] Ristroph K. D.; Feng J.; McManus S. A.; Zhang Y.; Gong K.; Ramachandruni H.; White C. E.; Prud’homme R. K. Spray Drying OZ439 Nanoparticles to Form Stable, Water-Dispersible Powders for Oral Malaria Therapy. J. Transl. Med. 2019, 17 (1), 97 10.1186/s12967-019-1849-8. 30902103 PMC6431012

[ref28] Caggiano N. J.; Wilson B. K.; Priestley R. D.; Prud’homme R. K. Development of an *In Vitro* Release Assay for Low-Density Cannabidiol Nanoparticles Prepared by Flash NanoPrecipitation. Mol. Pharmaceutics 2022, 19 (5), 1515–1525. 10.1021/acs.molpharmaceut.2c00041. 35412842

[ref29] Shin Etsu HPMCP Product Catalog; Shin-Etsu Chemical Co., Ltd., 2018.

[ref30] Shin-Etsu AQOAT Product Catalog; Shin-Etsu Chemical Co., Ltd., 2018.

[ref31] Donahue D. J.; Bartell F. E. The Boundary Tension at Water-Organic Liquid Interfaces. J. Phys. Chem. 1952, 56 (4), 480–484. 10.1021/j150496a016.

[ref32] Feng J.; Zhang Y.; McManus S. A.; Qian R.; Ristroph K. D.; Ramachandruni H.; Gong K.; White C. E.; Rawal A.; Prud’homme R. K. Amorphous Nanoparticles by Self-Assembly: Processing for Controlled Release of Hydrophobic Molecules. Soft Matter 2019, 15 (11), 2400–2410. 10.1039/C8SM02418A. 30776040

[ref33] Feng J.; Markwalter C. E.; Tian C.; Armstrong M.; Prud’homme R. K. Translational Formulation of Nanoparticle Therapeutics from Laboratory Discovery to Clinical Scale. J. Transl. Med. 2019, 17 (1), 200 10.1186/s12967-019-1945-9. 31200738 PMC6570894

[ref34] Yang B.; Wu C.; Ji B.; Ai X.; Kuang X.; Wu M.; Sun M.; Luo C.; He Z.; Sun J. The Biorelevant Concentration of Tween 80 Solution Is a Simple Alternative Medium to Simulated Fasted State Intestinal Fluid. RSC Adv. 2015, 5 (127), 104846–104853. 10.1039/C5RA17674C.

[ref35] Zoeller T.; Klein S. Simplified Biorelevant Media for Screening Dissolution Performance of Poorly Soluble Drugs. Dissolution Technol. 2007, 14 (4), 8–13. 10.14227/DT140407P8.

[ref36] Fotaki N.; Brown W.; Kochling J.; Chokshi H.; Miao H.; Tang K.; Gray V. Rationale for Selection of Dissolution Media: Three Case Studies. Dissolution Technol. 2013, 20 (3), 6–13. 10.14227/DT200313p6.

[ref37] Greenspan L. Humidity Fixed Points of Binary Saturated Aqueous Solutions. J. Res. Natl. Bur. Stand. Sect. Phys. Chem. 1977, 81A (1), 89 10.6028/jres.081A.011.

[ref38] Ward A. F. H.; Tordai L. Time-Dependence of Boundary Tensions of Solutions I. The Role of Diffusion in Time-Effects. J. Chem. Phys. 1946, 14 (7), 453–461. 10.1063/1.1724167.

[ref39] Fainerman V. B.; Makievski A. V.; Miller R. The Analysis of Dynamic Surface Tension of Sodium Alkyl Sulphate Solutions, Based on Asymptotic Equations of Adsorption Kinetic Theory. Colloids Surf. Physicochem. Eng. Asp. 1994, 87 (1), 61–75. 10.1016/0927-7757(94)02747-1.

[ref40] McClements D. J. Food Emulsions: Principles, Practices, and Techniques, third ed.; CRC Press, Taylor & Francis Group: Boca Raton, FL, 2016.

[ref41] Q3C—Tables and List Guidance for Industry; U.S. Food and Drug Administration, 2017.

[ref42] Pansare V. J.; Rawal A.; Goodwin A.; Beyerinck R.; Prud’homme R. K.; Friesen D. T.; Grass M.; Muske-Dukes A.; Vodak D. T. Millisecond Self-Assembly of Stable Nanodispersed Drug Formulations. Mol. Pharmaceutics 2018, 15 (2), 495–507. 10.1021/acs.molpharmaceut.7b00866. 29244515

[ref43] Noyes A. A.; Whitney W. R. THE RATE OF SOLUTION OF SOLID SUBSTANCES IN THEIR OWN SOLUTIONS. J. Am. Chem. Soc. 1897, 19 (12), 930–934. 10.1021/ja02086a003.

