# Supplementary material for: Formulation and Scale-up of Delamanid Nanoparticles via Emulsification for Oral Tuberculosis Treatment
Source: Mol Pharm. 2023 Aug 14;20(9):4546–58. doi: 10.1021/acs.molpharmaceut.3c00240 (PMC10481377; doi:10.1021/acs.molpharmaceut.3c00240)
Supplement: Supplementary file 1 — mp3c00240_si_001.pdf [file mp3c00240_si_001.pdf]

Formulation and scale-up of delamanid nanoparticles *via* emulsification for oral tuberculosis treatment

Nicholas J. Caggiano[1], Madeleine S. Armstrong[†,1], Joanna S. Georgiou[†,1], Aditya Rawal[2], Brian K. Wilson[1], Claire E. White[3,4], Rodney D. Priestley[1,5], and Robert K. Prud'homme[1,*]

[1]Department of Chemical and Biological Engineering, Princeton University, Princeton, New Jersey 08544, United States

[2]Mark Wainwright Analytical Centre, University of New South Wales, Sydney, NSW 2032, Australia

[3]Department of Civil and Environmental Engineering, Princeton University, Princeton, New Jersey 08544, United States

[4]Andlinger Center for Energy and the Environment, Princeton University, Princeton, New Jersey 08544, United States

[5]Princeton Materials Institute, Princeton University, Princeton, New Jersey 08544, United States

[*]Corresponding Author: prudhomm@princeton.edu; A301 Engineering Quadrangle, 41 Olden St, Princeton, NJ 08544, United States

[†]Contributed equally

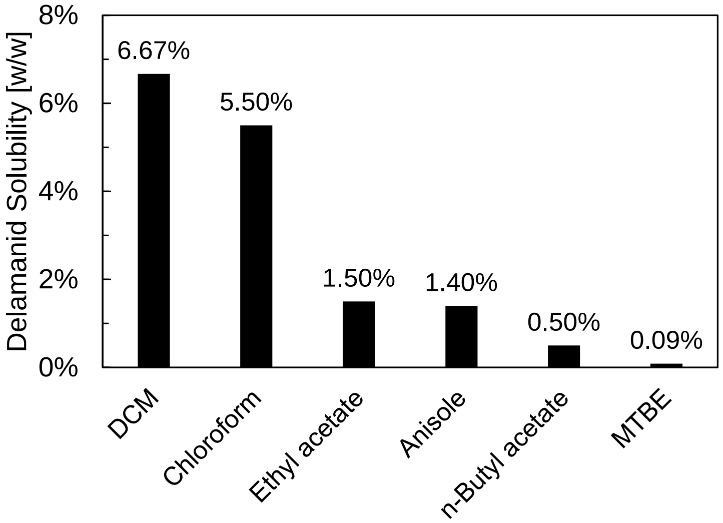

SI Figure 1. Solubility of delamanid in several organic, water-immiscible solvents: dichloromethane (DCM), chloroform, ethyl acetate, anisole, n-Butyl acetate, and methyl *tert*-butyl ether (MTBE). All data points were measured by thermogravimetric analysis (TGA) except for solubility in DCM.

For samples analyzed by TGA, crystalline delamanid was added to organic solvent at approximately 200 mg mL^{-1}, in excess of its expected solubility. After gentle vortexing, each vial was allowed to sit at room temperature overnight for approximately 12 hours, after which 100 μL of supernatant was removed for thermogravimetric analysis (TGA). Samples were analyzed using a Q50 TGA (TA Instruments, New Castle, DE). Under a nitrogen atmosphere, each sample was heated at a rate of 5 °C min^{-1} from room temperature to 120 °C, held isothermally for 20 min, and then cooled at 5 °C min^{-1} to room temperature. The residual solids mass was measured at the end of the isothermal step, and the solubility of delamanid was calculated as the ratio of the residual solids mass to the mass of the sample at t=0. Analysis was performed using the TA Instruments Universal Analysis software package.

Solubility in DCM was measured by adding a known mass of delamanid to a vial and gradually adding DCM volumetrically, stirring between additions. When all delamanid was visually solubilized, the final volume of DCM added was recorded and the solubility reported as a weight percent.

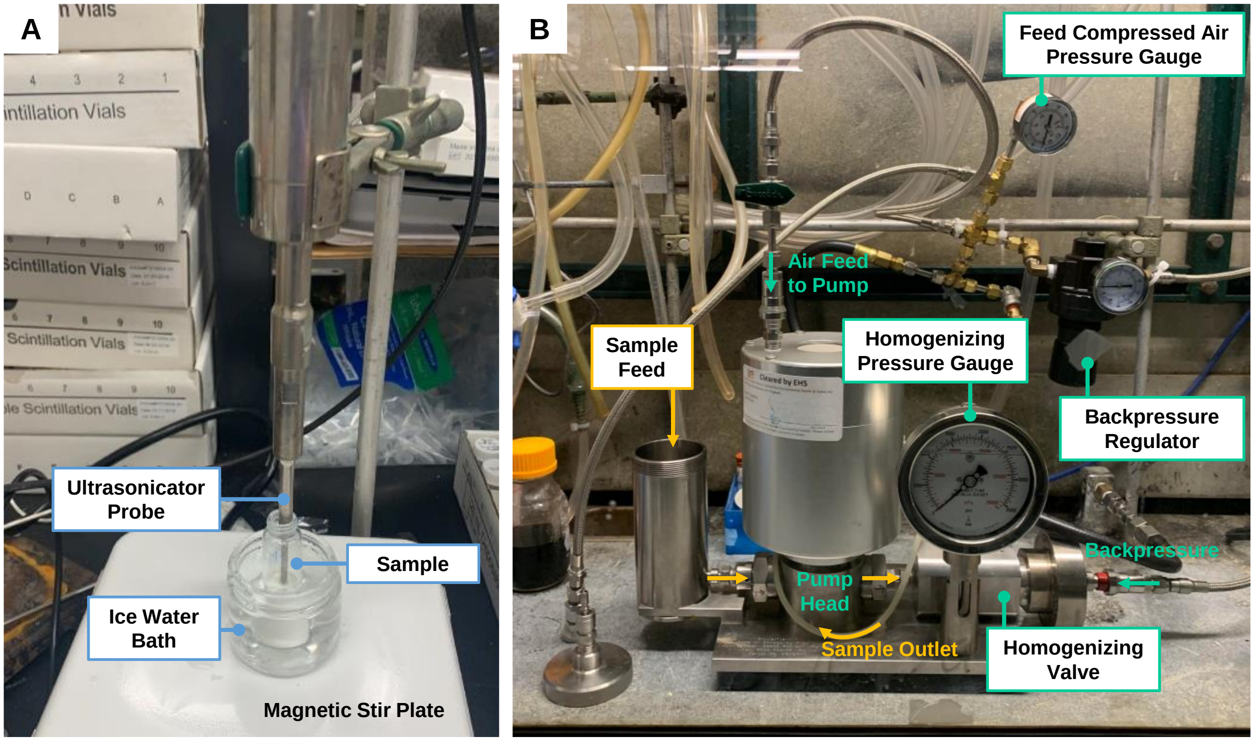

SI Figure 2. Images of experimental setups for **A.** probe-tip ultrasonication (VibraCell™ VC-50) and **B.** high-pressure homogenization (EmulsiFlex C5). During all experiments, the homogenizer was immersed in a room temperature water bath (T_{bath} ~ 20 °C) such that the homogenizing valve assembly was entirely submerged.

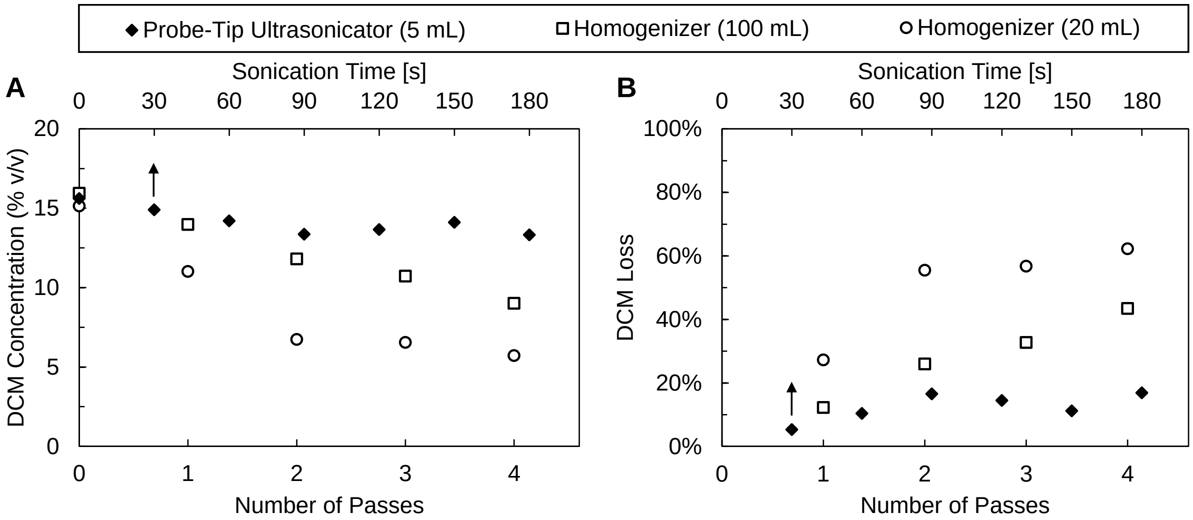

SI Figure 3. Dichloromethane (DCM) concentration (**A**) and corresponding loss percentage (**B**) as a function of homogenizer passes or ultrasonication time for blank DCM / water samples. Samples were prepared containing only 15% DCM and 85% water by volume, as the presence of drug and stabilizer interfered with quantification of residual DCM by HPLC. The amount of DCM lost during emulsification is important to quantify because significant loss of solvent in the system can lead to drug precipitation during emulsification.

For samples emulsified by high-pressure homogenization, the percentage of DCM lost was lower at higher batch size. The fact that DCM loss did not increase with increased batch size suggests that the mass of DCM loss may have a fixed component. Therefore, the effects of DCM loss may decrease at larger scales. Samples emulsified by probe-tip ultrasonication experienced comparatively minimal loss of DCM (<20%) over the 3 minute sonication duration.

For samples emulsified by probe-tip ultrasonication, 7 discrete samples of 5 mL each were prepared; each was sonicated for 0, 30, 60, 120, or 180 seconds in an ice water bath using the same conditions under which the delamanid emulsions were prepared. For high-pressure homogenization, sample volumes of 20 mL and 100 mL were tested. For each set of experiments, 5 discrete samples were prepared and homogenized for 0, 1, 2, 3 or 4 sequential passes using the same conditions as the delamanid emulsions. The Avestin EmulsiFlex C5 homogenizer was immersed in a room temperature water bath for the duration of all experiments.

Following emulsification, samples were volumetrically diluted 1:1 with methanol to form a single liquid phase. Reverse-phase HPLC (Phenomenex Kinetex C18, 100 Å, 150 x 4.6 mm, 5 μm particles) with an Agilent 1100 series HPLC was used to quantify the amount of DCM in each sample, C_{sample}. DCM was eluted (RT = 2.8 min) with an isocratic mobile phase of 50:50 acetonitrile/water with 0.05% trifluoroacetic acid at a flow rate of 1 mL min^{-1} and column temperature of 35 °C, with UV detection at 220 nm. A linear standard curve was constructed for DCM concentrations between 0.5 and 10% v/v DCM in methanol (**SI Figure 4**). The concentration of DCM in the sample that did not undergo emulsification was termed C_0. The DCM Loss percentage was calculated as $100 \times C_{sample} / C_0$.

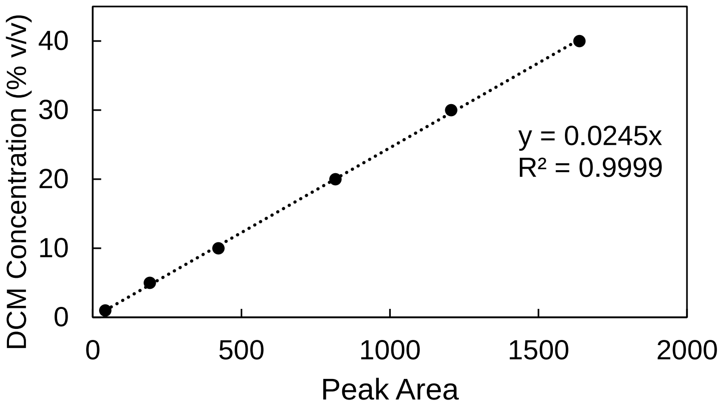

SI Figure 4. Exemplary HPLC standard curve for dichloromethane (DCM). Samples of known concentrations were prepared in methanol. The UV absorbance was highly linear with DCM concentration in the range of 1 – 40% v/v, and a linear regression yielded $C_{\mathrm{DCM}} = 0.0245(\text{Peak Area})$, where C_{DCM} is the concentration of DCM contained in the sample (as % v/v), and Peak Area is the integrated area of the UV absorbance peak at 220 nm. All samples were diluted into this concentration range for HPLC analysis.

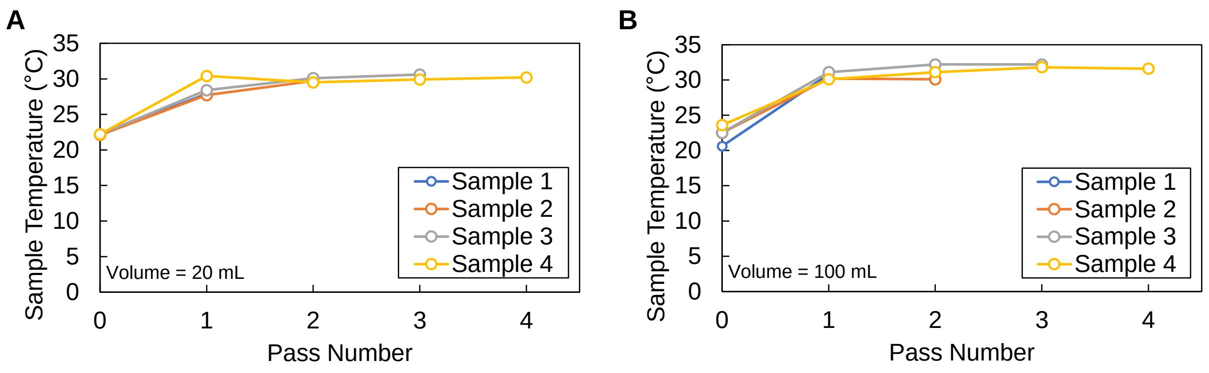

SI Figure 5. Sample temperature as a function of pass number for high-pressure homogenized samples from **SI Figure 3** for batch sizes of 20 mL **(A)** and 100 mL **(B)**. Sample 1 refers to the sample homogenized for one complete pass, Sample 2 for two passes, etc. All samples displayed similar temperature profiles, starting from room temperature of approx. 22 °C and plateauing in the range of 30 – 32 °C. Tabulated values are reported in **SI Table 1**.

SI Table 1. Tabulated temperature values (°C) for samples described in **SI Figure 5**.

	Batch volume (mL)			20	Batch volume (mL)			100
	Bath temp (°C)			20.2	Bath temp (°C)			21.4
**Pass Number**	**Sample 1**	**Sample 2**	**Sample 3**	**Sample 4**	**Sample 1**	**Sample 2**	**Sample 3**	**Sample 4**
0	22.1	22.1	22.2	22.2	20.6	22.5	22.5	23.6
1	27.9	27.7	28.4	30.4	30.8	30.2	31.1	30.1
2		29.7	30.1	29.5		30.1	32.2	31.1
3			30.6	29.9			32.2	31.8
4				30.2				31.6

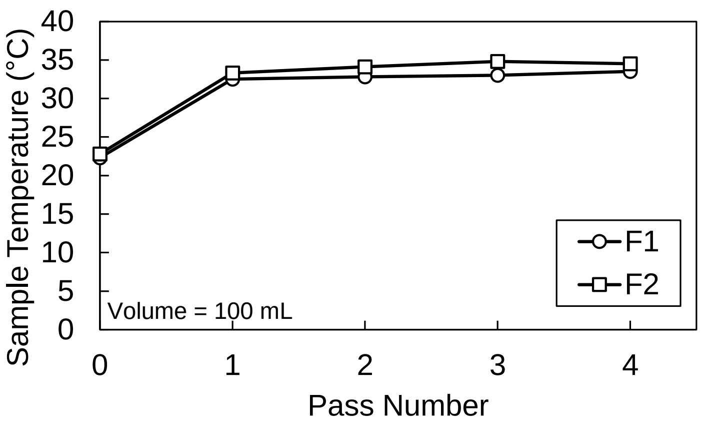

SI Figure 6. Sample temperature as a function of homogenization pass number for emulsion formulations F1 and F2 with a 1:1 mass ratio of HPMC to lecithin used as stabilizer. Samples were prepared at a batch size of 100 mL. Samples were initially at room temperature and increased to 32 – 33 °C after one pass, where they remained approximately plateaued for all successive homogenization passes. The Avestin EmulsiFlex C5 homogenizer was immersed in a room temperature water bath for the duration of all experiments. Tabulated values are reported in **SI Table 2**.

SI Table 2. Tabulated temperature values (°C) for samples described in **SI Figure 6**.

**Formulation**	**F1**	**F2**
Batch Volume (mL)	100	100
Bath temperature (°C)	22.9	23.7
**Pass Number**	**Sample Temperature (°C)**	**Sample Temperature (°C)**
0	22.3	22.8
1	32.5	33.3
2	32.8	34.1
3	33.0	34.8
4	33.5	34.5

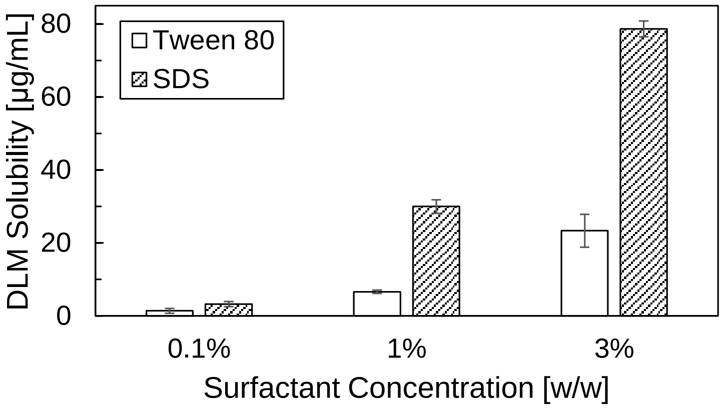

SI Figure 7. Solubility of delamanid (DLM) in 150 mM pH 7 HEPES buffer with added Tween 80 or sodium dodecyl sulfate (SDS) surfactant, measured by HPLC. The Tween 80 surfactant is nonionic, while the SDS is anionic. A saturated solution of 100 mg mL^{-1} DLM in THF was spiked into the aqueous buffer (100 µL THF into 10 mL buffer) to create a supersaturated solution. Samples were incubated at 37 °C overnight, after which they were centrifuged (21,000 *g*, 10 min) to pellet undissolved DLM. Samples were diluted with solvent as necessary and the concentration of dissolved DLM was quantified by HPLC.

HEPES buffer with 3% added Tween 80 was selected for use as *in vitro* dissolution media for two reasons. First, the solubility of DLM in HEPES with 3% Tween 80 is sufficient to enable higher DLM dosing in the *in vitro* studies (approx. 10 – 15 µg/mL), enabling more accurate detection and quantification of DLM by HPLC (LLOQ = 0.7 µg/mL). Second, although SDS seems to provide enhanced solubility of DLM relative to Tween 80, the tabulated HPLC retention time data suggests that DLM and SDS may form an electrostatic complex. To avoid this electrostatic interaction, the nonionic Tween 80 was used, as it serves only as a micellar sink into which the hydrophobic DLM partitions.

SI Table 3. Tabulated solubility and HPLC retention time of delamanid (DLM) in 150 mM pH 7 HEPES buffer with added Tween 80 or sodium dodecyl sulfate (SDS) surfactant.

Surfactant	Surfactant Concentration (w/w)	Delamanid Solubility (µg/mL)	Delamanid HPLC Retention Time (min)
Tween 80	0.1%	1.88 ± 0.68	7.2
	1%	7.06 ± 0.43	7.2
	3%	23.6 ± 4.44	7.2
SDS	0.1%	3.73 ± 0.69	7.5
	1%	30.2 ± 1.82	9.5
	3%	78.4 ± 2.17	10

SI Table 4. Tabulated solubility data for delamanid (DLM) in commercially available biorelevant media obtained from Biorelevant.com, Ltd. (London, United Kingdom).

	**Delamanid solubility (ug mL^{-1})**	
**Buffer**	Unfiltered	Filtered
Fasted State Gastric Fluid (FaSSGF)	15.6	3.75
Fasted State Intestinal Fluid (FaSSIF)	0.861	0.897
Fed State Intestinal Fluid (FeSSIF)	6.16	2.86

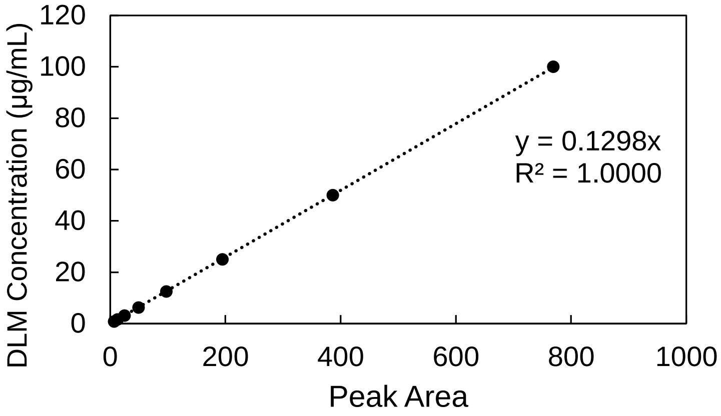

SI Figure 8. Exemplary HPLC standard curve for delamanid (DLM). Samples of known concentrations were prepared in tetrahydrofuran. The UV absorbance was highly linear with DLM concentration in the range of 1 – 50 μg/mL, and a linear regression yielded $C_{\mathrm{DLM}} = 0.1298(\text{Peak Area})$, where C_{DLM} is the concentration of DLM contained in the sample (in μg/mL), and Peak Area is the integrated area of the UV absorbance peak at 330 nm. The lower limit of quantification (LLOQ) was 0.7 μg/mL. All samples were diluted into this concentration range for HPLC analysis.

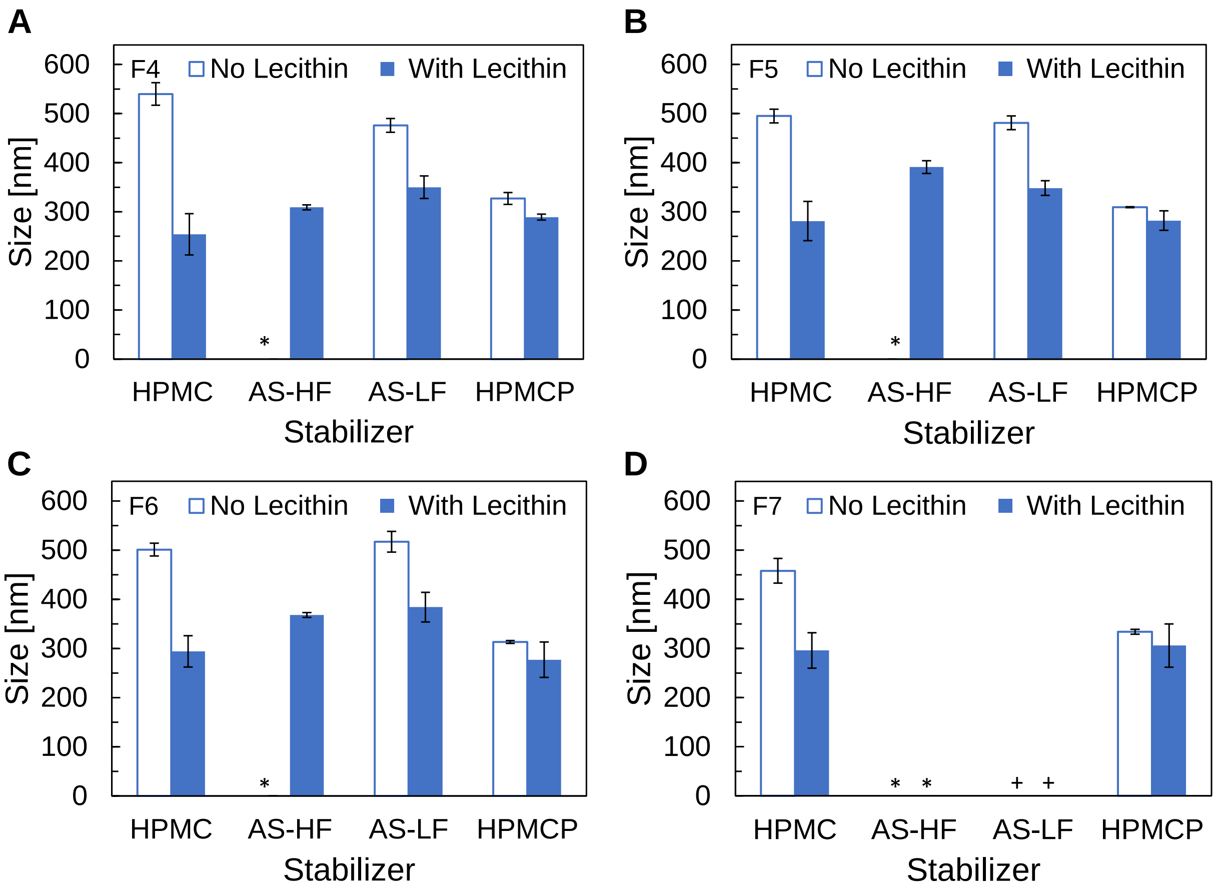

SI Figure 9. Z-average hydrodynamic diameter of emulsion formulations **A)** F4, **B)** F5, **C)** F6, and **D)** F7 prepared by probe-tip ultrasonication using HPMC, HPMCAS-HF, HPMCAS-LF, or HPMCP as stabilizer (open bars) or a 1:1 mass ratio of lecithin to each of the cellulosic polymers (filled bars). The * indicates that the formulation was not prepared due to solubility limitations of the cellulosic stabilizer in the aqueous phase. The + indicates that the formulation gelled upon emulsification.

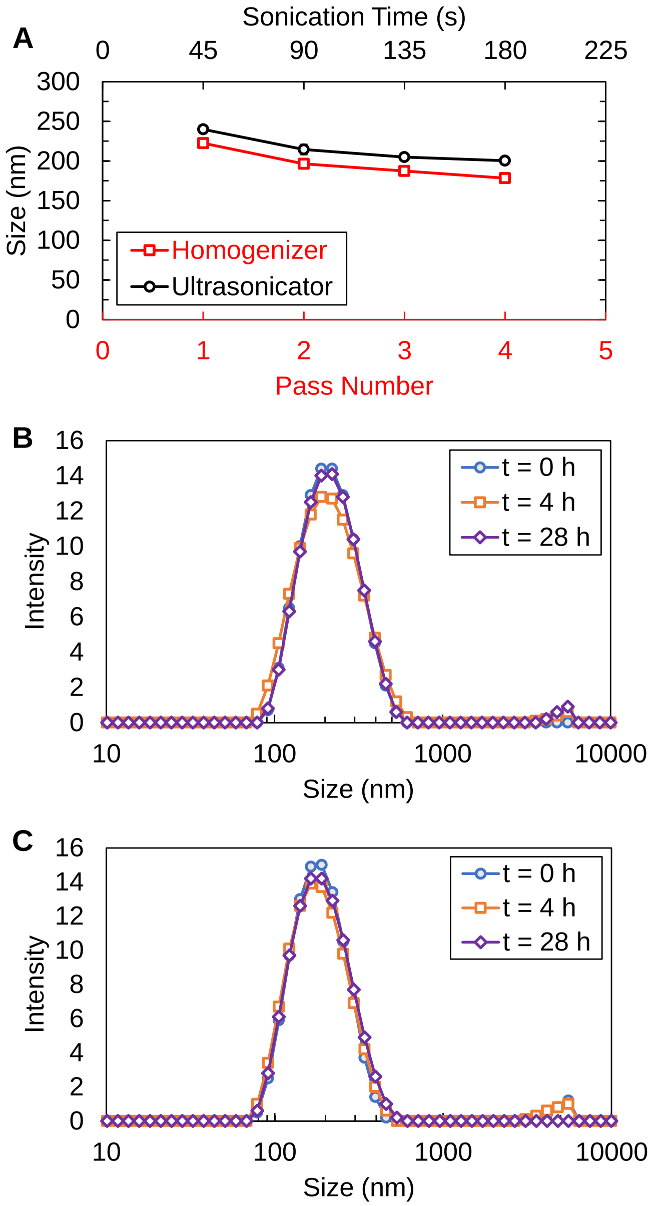

SI Figure 10. A) Z-average hydrodynamic diameter of PCL core / HPMC-lecithin stabilized F1 formulation as a function of homogenizer pass number (for formulation prepared by high-pressure homogenization) or sonication time (for formulation prepared by probe-tip ultrasonication); **B)** Particle size distributions over time of PCL core / HPMC-lecithin formulation prepared by high pressure homogenization (4 passes), measured by DLS; **C)** Particle size distributions over time of PCL core / HPMC-lecithin formulation prepared by probe-tip ultrasonication (180 s soncation time), measured by DLS. The particle size distributions across all timepoints are similar between formulations prepared by homogenization and ultrasonication, illustrating that the formulations exhibit good size stability over time and that both processing techniques can be used to produce nearly identical particles at different scales. As expected, due to the higher energy input of homogenization, particles produced by homogenization exhibited slightly smaller average size than those produced by ultrasonication.

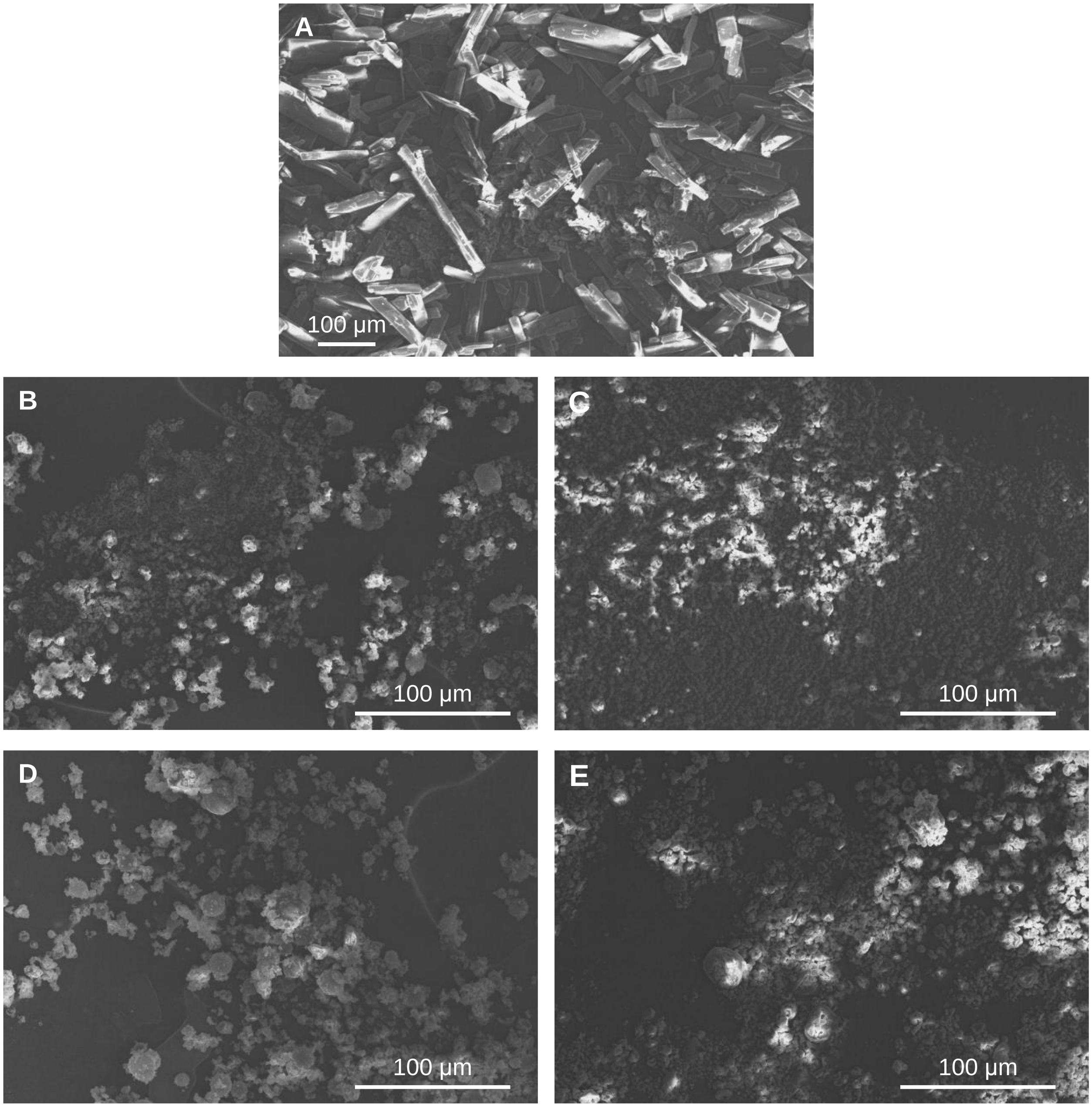

SI Figure 11. SEM micrographs of **A)** delamanid crystals, **B)** F2 DLM/HPMC-lecithin spray-dried emulsion formulation at t=0, **C)** F2 DLM/HPMC-lecithin spray-dried emulsion after 4 weeks of accelerated stability testing (50 °C / 75% RH), **D)** DLM/HPMCP spray-dried solid dispersion at t=0, **E)** DLM/HPMCP spray-dried solid dispersion after 4 weeks of accelerated stability testing (50 °C / 75% RH). Micrographs were obtained using an FEI Verios 460 SEM at 10 KV acceleration voltage. All formulations exhibit a wrinkled sphere morphology due to the formation of a dried skin at the droplet surface during drying. No evidence of DLM crystallites was observed visually by SEM, confirming the need for the other characterization techniques applied in this work (XRD, DSC, ssNMR).

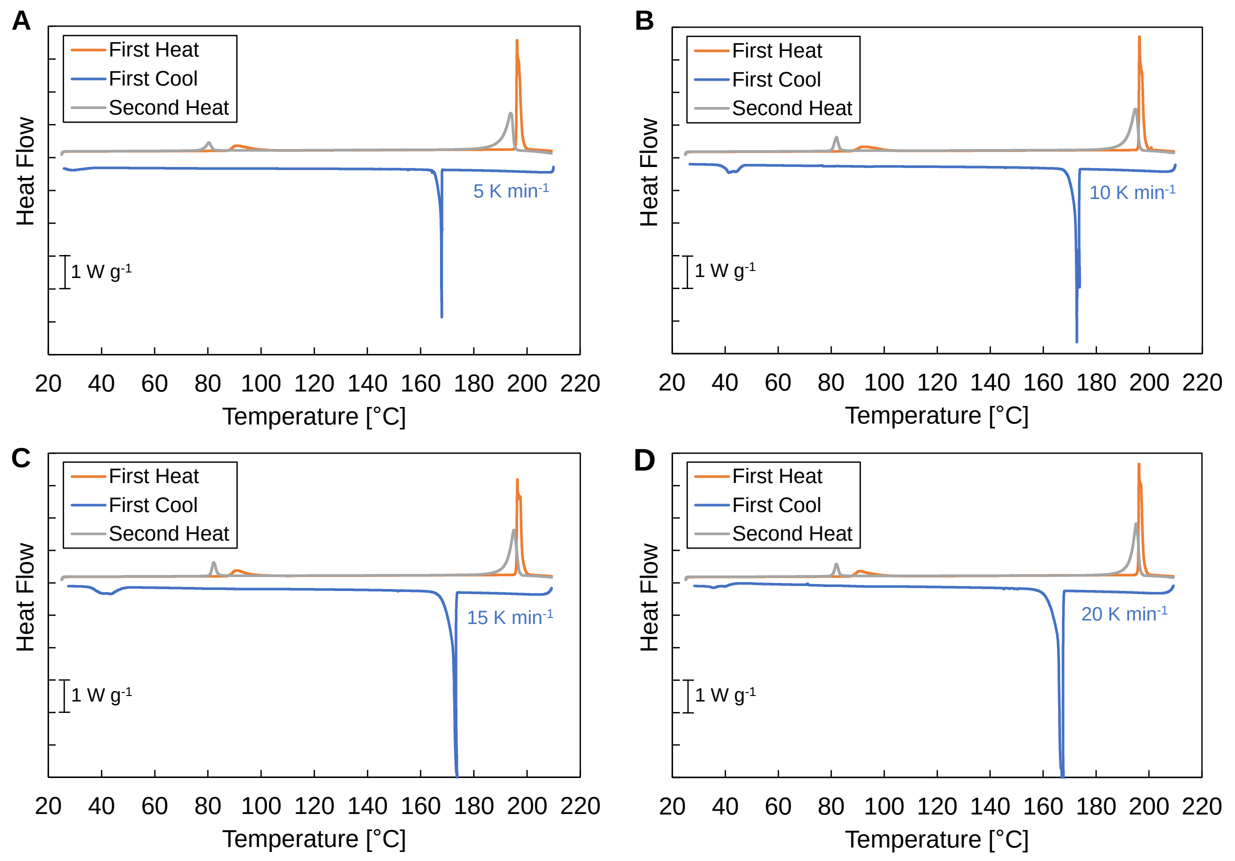

SI Figure 12. Recrystallization studies of pure delamanid performed by DSC showing recrystallization from the melt state at cooling rates of **A)** 5 K min^{-1}, **B)** 10 K min^{-1}, **C)** 15 K min^{-1}, and **D)** 20 K min^{-1}. Such recrystallization behavior highlights the propensity of delamanid to recrystallize and would place it in Class I as defined by Baird *et al.* (*J. Pharm. Sci.* **2010**, *99* (9), 3787-3806).

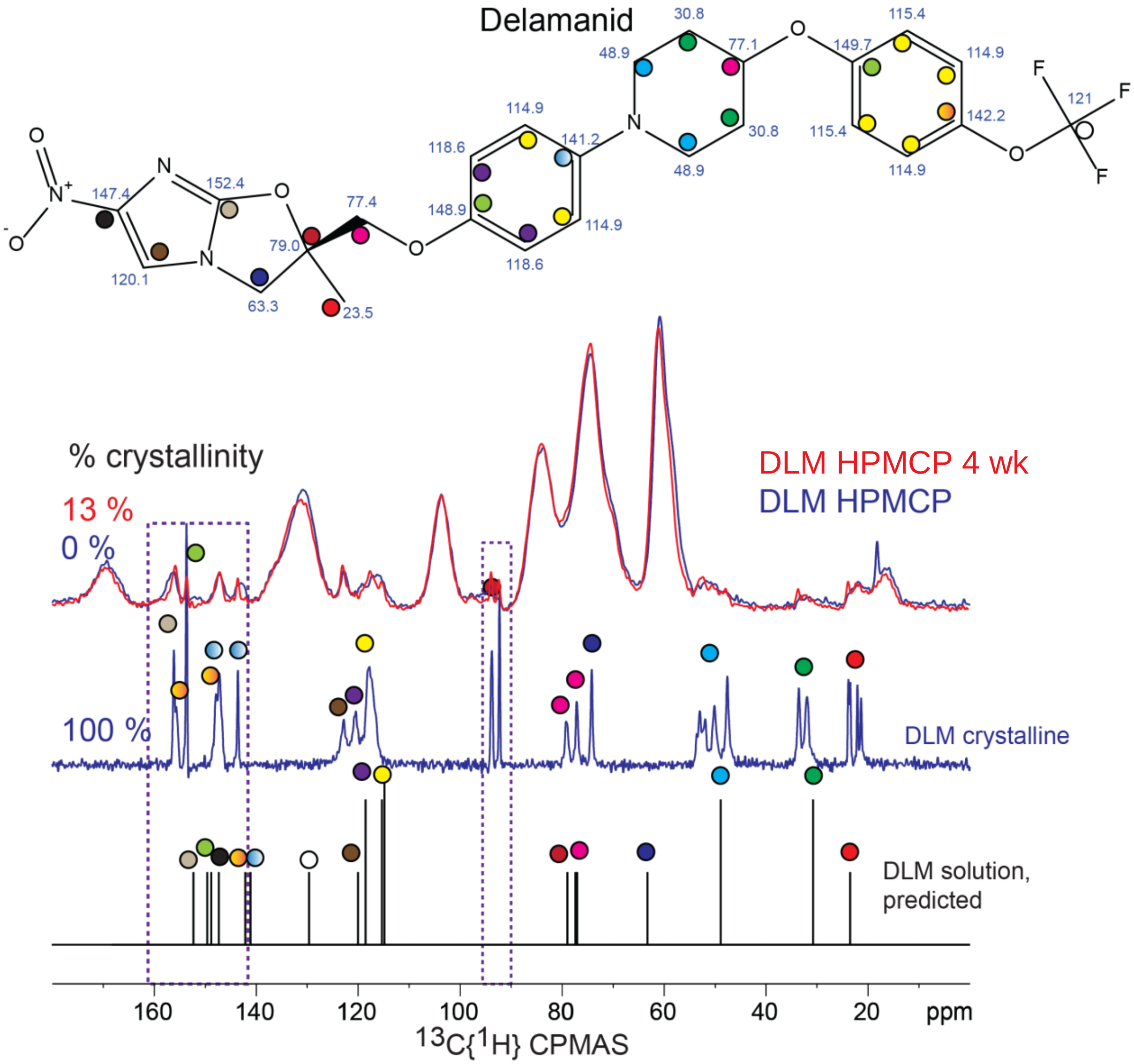

SI Figure 13. ^{13}C solid state NMR spectra of the DLM/HPMCP formulation before and after 4 weeks of open-vial storage at 50 °C / 75% RH, zoomed in on selected regions highlighting the DLM-only peaks. The estimated (lower limit) percent crystallinity of DLM in the different formulations is annotated on the left (calculated with respect to the mass of DLM in each formulation). The 4-week stability tested samples (open vial, 50 °C / 75% RH) are plotted in red and overlayed on the respective initial formulations. The dashed rectangles highlight the specific regions where the DLM signals are nominally well resolved and not overlapped by signals of the excipients.

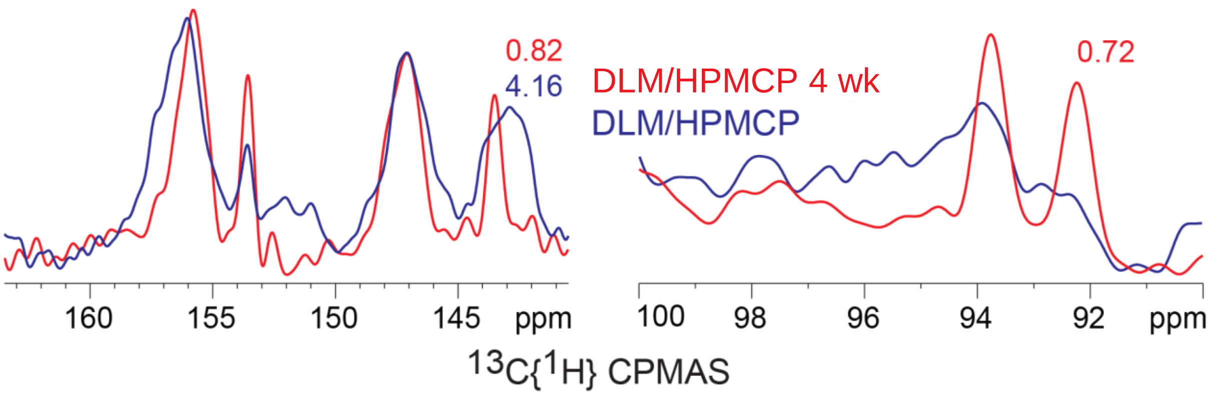

SI Figure 14. ^{13}C solid state NMR spectra of the fresh (blue) and 4-week open vial, 50 °C / 75% RH (red) DLM HPMCP ASD formulation zoomed in on selected regions highlighting the DLM only peaks. The change in peak linewidths of selected DLM peaks at 143.63 ppm and 92.32 ppm are highlighted next to the peaks.

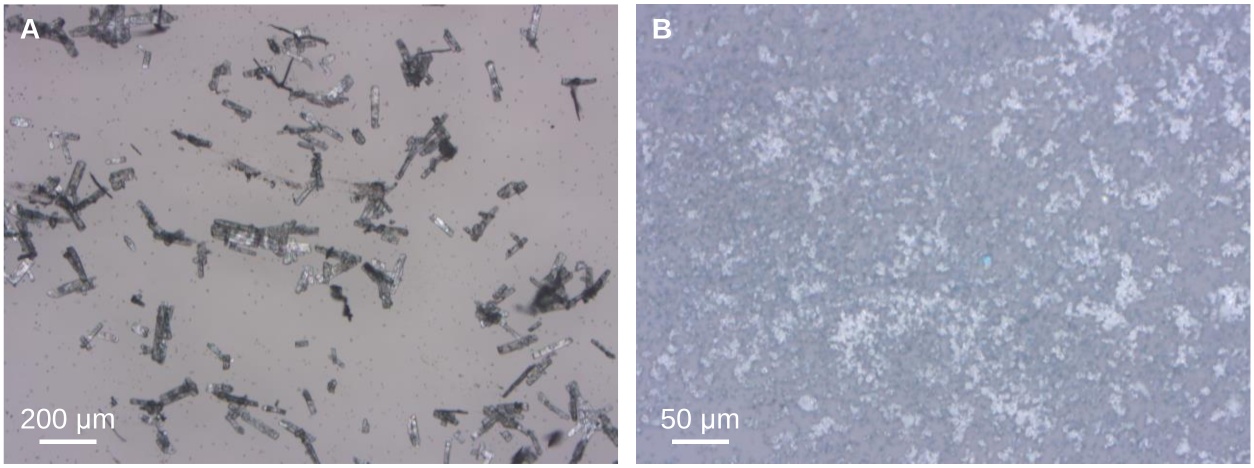

SI Figure 15. A) DLM crystals as received from the manufacturer, **B)** DLM spray-dried from a dichloromethane solution without addition of additional excipients.

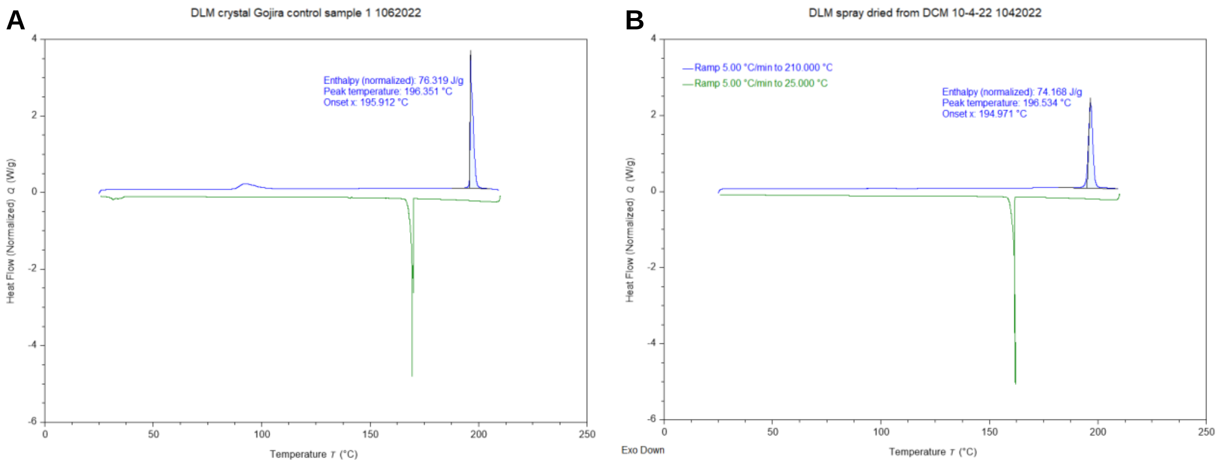

SI Figure 16. A) Differential scanning calorimetry (DSC) thermogram of DLM crystals as received from the manufacturer with melting enthalpy (on first heating) calculated by peak integration, **B)** DSC thermogram of DLM spray-dried from a dichloromethane solution (without addition of additional excipients crystals) with melting enthalpy (on first heating) calculated by peak integration. The ratio of the enthalpies indicates that the spray-dried material is 97% crystalline. Heating and cooling rates were 5 °C min^{-1}.

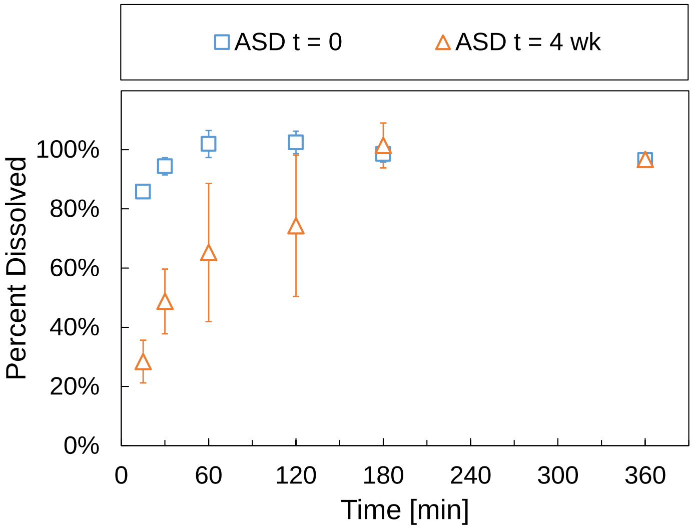

SI Figure 17. *In vitro* dissolution profile for an amorphous solid dispersion (ASD) formulation containing 20% DLM and 80% HPMCP (HP-50), prepared by spray drying from a 1:1 volumetric mixture of methanol and dichloromethane. Initially at t = 0 the ASD displayed fairly rapid and complete dissolution kinetics; however, after 4 weeks of storage at 50 °C / 75% RH the *in vitro* dissolution performance was significantly reduced.

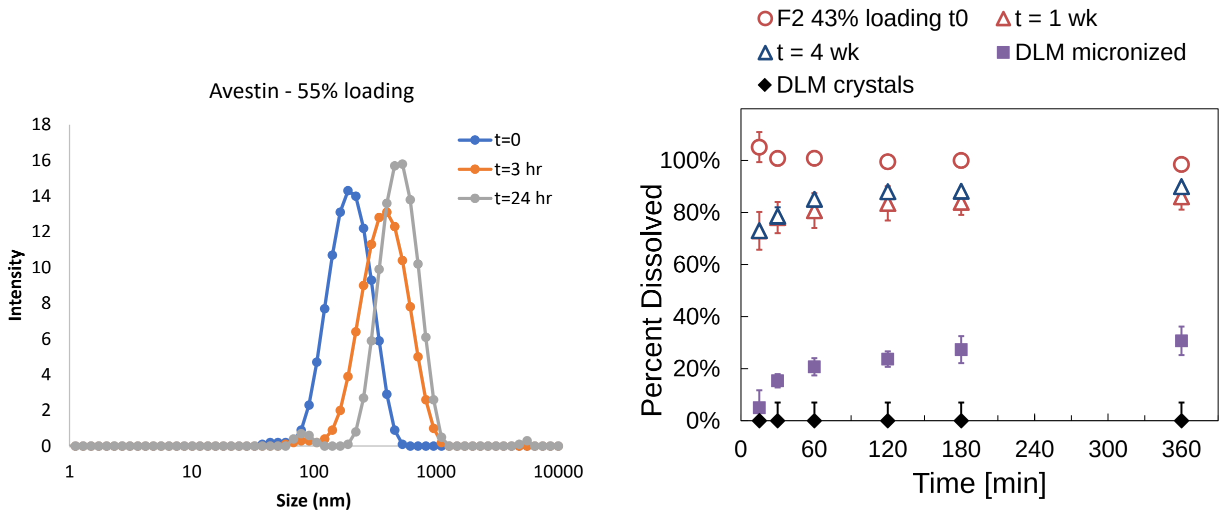

SI Figure 18. A) Particle size distributions over time (t = 0, 3, 24 h) for higher-loaded emulsion formulation containing 55% DLM by mass: 0.67 wt% DLM, 0.5 wt% lecithin, and 0.037 wt% HPMC (a 13.5:1 mass ratio of lecithin to HPMC). The dispersed phase was 15% v/v dichloromethane. **B)** *In vitro* dissolution profile for the higher-loaded emulsion formulation spray-dried, with 0.41:1 mass ratio of HPMC to nanoparticles added as a bulking agent. The resulting powder contained 43% DLM. The 43% DLM-loaded sample displayed rapid dissolution kinetics similar to those displayed by the 20% DLM-loaded sample. Dissolution kinetics for the as-received crystalline material and micronized crystalline DLM (prepared by spray drying from solution) as included for comparison.

APPENDIX A. PROBE-TIP ULTRASONICATION EMULSION SCREENING SIZE DATA

SI Table A1. Average emulsion droplet size over time for emulsions stabilized by HPMC or a mixture of HPMC and lecithin (1:1 mass ratio)

**DLM HPMC without Lecithin** (diameter in nm)			
**Formulation**	**t = 0 h**	**t ~ 3 h**	**t ~ 24 h**
F1	479 ± 2	495 ± 2	655 ± 29
F2	499 ± 24	534 ± 17	610 ± 27
F3	491 ± 37	446 ± 8	645 ± 21
F12	557 ± 24	572 ± 13	598 ± 29
F4	540 ± 23	562 ± 8	604 ± 19
F5	495 ± 14	520 ± 14	582 ± 4
F6	501 ± 13	346 ± 18	560 ± 11
F7	458 ± 25	346 ± 18	533 ± 9
**DLM HPMC with Lecithin** (diameter in nm)			
**Formulation**	**t = 0 h**	**t ~ 3 h**	**t ~ 24 h**
F1	251 ± 2	276 ± 6	292 ± 6
F2	252 ± 36	278 ± 6	305 ± 13
F3	281 ± 35	290 ± 5	350 ± 11
F12	292 ± 9	307 ± 7	316 ± 21
F4	254 ± 42	301 ± 6	328 ± 18
F5	281 ± 40	307 ± 6	302 ± 31
F6	294 ± 32*	469 ± 87*	335 ± 16
F7	296 ± 36	308 ± 5	342 ± 29

*DLS cumulant fits diverged from data, indicating PSD and Z-average may not accurately represent the particle size

SI Table A2. Average emulsion droplet size over time for emulsions stabilized by HPMCAS HG or a mixture of HPMCAS HG and lecithin (1:1 mass ratio)

**LM HPMCAS HG without Lecithin** (diameter in nm)			
**Formulation**	**t = 0 h**	**t ~ 3 h**	**t ~ 24 h**
F1	361 ± 21	470 ± 9	478 ± 30
F2	312 ± 4	392 ± 8	447 ± 20
F3	258 ± 14	394 ± 2	476 ± 20
F12	N/A*	N/A*	N/A*
F4	N/A*	N/A*	N/A*
F5	N/A*	N/A*	N/A*
F6	N/A*	N/A*	N/A*
F7	N/A*	N/A*	N/A*
**DLM HPMCAS HG with Lecithin** (diameter in nm)			
**Formulation**	**t = 0 h**	**t ~ 3 h**	**t ~ 24 h**
F1	338 ± 4	401 ± 6	454 ± 34
F2	287 ± 9	372 ± 6	395 ± 29
F3	274 ± 17	348 ± 16	364 ± 29
F12	317 ± 2	394 ± 10	450 ± 34
F4	309 ± 5	400 ± 4	352 ± 17
F5	391 ± 13	452 ± 7	529 ± 43
F6	368 ± 5	457 ± 6	536 ± 48
F7	N/A*	N/A*	N/A*

*Not prepared due to HPMCAS solubility limitations

SI Table A3. Average emulsion droplet size over time for emulsions stabilized by HPMCAS LG or a mixture of HPMCAS LG and lecithin (1:1 mass ratio)

**DLM HPMCAS LG without Lecithin** (diameter in nm)			
**Formulation**	**t = 0 h**	**t ~ 3 h**	**t ~ 24 h**
F1	432 ± 13	587 ± 4	792 ± 9
F2	516 ± 11	633 ± 9	837 ± 14
F3	499 ± 23	621 ± 25	1194 ± 131
F12	499 ± 6	570 ± 11	817 ± 70
F4	476 ± 14	572 ± 13	806 ± 26
F5	481 ± 14	557 ± 22	768 ± 47
F6	517 ± 21	586 ± 8	827 ± 124
F7	N/A*	N/A*	N/A*
**DLM HPMCAS LG with Lecithin** (diameter in nm)			
**Formulation**	**t = 0 h**	**t ~ 3 h**	**t ~ 24 h**
F1	315 ± 14	397 ± 7	529 ± 29
F2	335 ± 14	415 ± 3	540 ± 9
F3	340 ± 4	468 ± 6	607 ± 42
F12	316 ± 2	408 ± 5	497 ± 27
F4	350 ± 23	433 ± 21	497 ± 8
F5	348 ± 15	408 ± 2	508 ± 24
F6	384 ± 30	463 ± 19	652 ± 40
F7	N/A*	N/A*	N/A*

*Gelled during sonication

SI Table A4. Average emulsion droplet size over time for emulsions stabilized by HPMCP (HP-50) or a mixture of HPMCP and lecithin (1:1 mass ratio)

**DLM HPMCP without Lecithin** (diameter in nm)			
**Formulation**	**t = 0 h**	**t ~ 3 h**	**t ~ 24 h**
F1	323 ± 8	426 ± 6	584 ± 14
F2	298 ± 5	423 ± 6	615 ± 8
F3	280 ± 7	412 ± 8	600 ± 12
F12	321 ± 8	413 ± 8	609 ± 12
F4	327 ± 12	382 ± 10	649 ± 7
F5	309 ± 1	406 ± 13*	654 ± 25
F6	313 ± 3	360 ± 10*	591 ± 28
F7	334 ± 5	402 ± 16	626 ± 14
**DLM HPMCP with Lecithin** (diameter in nm)			
**Formulation**	**t = 0 h**	**t ~ 3 h**	**t ~ 24 h**
F1	234 ± 25	328 ± 7	441 ± 24
F2	232 ± 6	305 ± 13	445 ± 13
F3	272 ± 4	359 ± 14	521 ± 6
F12	249 ± 23	303 ± 28	438 ± 6
F4	289 ± 6	325 ± 4	436 ± 11
F5	282 ± 20	311 ± 9	445 ± 3
F6	277 ± 36	274 ± 4	465 ± 2
F7	306 ± 44	322 ± 18	490 ± 16

APPENDIX B. PENDANT DROP TENSIOMETRY REGRESSION DATA

Tensiometry measurements were performed using a pendant drop of dichloromethane and an external phase of dichloromethane-saturated water. The stabilizer was dissolved in the water phase. A linear regression was performed for each measurement using an infinite-time asymptotic solution to the Ward and Tordai model for modeling adsorption of surfactant to a non-deforming surface:

$$\gamma = \gamma_\infty + \frac{RT\Gamma_\infty^2}{C_0}\sqrt{\frac{\pi}{4Dt}}$$

SI Table B1. Tabulated values of equilibrium interfacial tension, γ_∞, for a pendant dichloromethane drop in an external phase of dichloromethane-saturated water in the presence of stabilizer.

**Stabilizer**	**Concentration (w/w)**	**IFT, γ_∞ (mN/m)**
none (control)	-	27.9 ± 0.6
HPMCP (HP-50)	0.001%	24.8 ± 2.0
	0.010%	24.1 ± 0.6
	0.050%	10.5 ± 0.8
	0.100%	8.8 ± 0.6
HPMC	0.001%	4.9 ± 0.5
	0.010%	1 ± 0.9
	0.050%	2.1 ± 0.7
	0.100%	1.2 ± 0.7
HPMCAS-LF	0.001%	22.8 ± 0.7
	0.010%	15.4 ± 0.7
	0.050%	11.9 ± 0.3
	0.100%	8.7 ± 1.9
HPMCAS-HF	0.010%	17.8 ± 0.9
	0.050%	12.7 ± 0.2
	0.100%	9.6 ± 0.2
